# Optimizing Peripheral Nerve Regeneration: Surgical Techniques, Biomolecular and Regenerative Strategies—A Narrative Review

**DOI:** 10.3390/ijms26083895

**Published:** 2025-04-20

**Authors:** Andreea Grosu-Bularda, Cristian-Vladimir Vancea, Florin-Vlad Hodea, Andrei Cretu, Eliza-Maria Bordeanu-Diaconescu, Catalina-Stefania Dumitru, Vladut-Alin Ratoiu, Razvan-Nicolae Teodoreanu, Ioan Lascar, Cristian-Sorin Hariga

**Affiliations:** 1 Department 11, Discipline Plastic and Reconstructive Surgery, University of Medicine and Pharmacy Carol Davila, 050474 Bucharest, Romania; andreea.grosu-bularda@umfcd.ro (A.G.-B.); cristian.hariga@umfcd.ro (C.-S.H.); 2 Clinic of Plastic Surgery and Reconstructive Microsurgery, Clinical Emergency Hospital of Bucharest, 014461 Bucharest, Romania

**Keywords:** nerve injury, peripheral nerve regeneration, nerve repair, autograft, allograft, conduits

## Abstract

Peripheral nerve injury disrupts the function of the peripheral nervous system, leading to sensory, motor, and autonomic deficits. While peripheral nerves possess an intrinsic regenerative capacity, complete sensory and motor recovery remains challenging due to the unpredictable nature of the healing process, which is influenced by the extent of the injury, age, and timely intervention. Recent advances in microsurgical techniques, imaging technologies, and a deeper understanding of nerve microanatomy have enhanced functional outcomes in nerve repair. Nerve injury initiates complex pathophysiological responses, including Wallerian degeneration, macrophage activation, Schwann cell dedifferentiation, and axonal sprouting. Complete nerve disruptions require surgical intervention to restore nerve continuity and function. Direct nerve repair is the gold standard for clean transections with minimal nerve gaps. However, in cases with larger nerve gaps or when direct repair is not feasible, alternatives such as autologous nerve grafting, vascularized nerve grafts, nerve conduits, allografts, and nerve transfers may be employed. Autologous nerve grafts provide excellent biocompatibility but are limited by donor site morbidity and availability. Vascularized grafts are used for large nerve gaps and poorly vascularized recipient beds, while nerve conduits serve as a promising solution for smaller gaps. Nerve transfers are utilized when neither direct repair nor grafting is possible, often involving re-routing intact regional nerves to restore function. Nerve conduits play a pivotal role in nerve regeneration by bridging nerve gaps, with significant advancements made in material composition and design. Emerging trends in nerve regeneration include the use of 3D bioprinting for personalized conduits, gene therapy for targeted growth factor delivery, and nanotechnology for nanofiber-based conduits and stem cell therapy. Advancements in molecular sciences have provided critical insights into the cellular and biochemical mechanisms underlying nerve repair, leading to targeted therapies that enhance axonal regeneration, remyelination, and functional recovery in peripheral nerve injuries. This review explores the current strategies for the therapeutic management of peripheral nerve injuries, highlighting their indications, benefits, and limitations, while emphasizing the need for tailored approaches based on injury severity and patient factors.

## 1. Overview of Peripheral Nerve Injuries

### 1.1. Introduction

Peripheral nerve injury is defined as damage or dysfunction of the peripheral nervous system, which is responsible for transmitting motor, sensory, and autonomic signals between the central nervous system and the rest of the body. The incidence of peripheral nerve injuries varies considerably in clinical studies, with the reported prevalence ranging from 0.13% to 5%, depending on the population studied and the underlying etiology [1,2].

Peripheral nerve injuries result from a range of mechanical, ischemic, inflammatory, or degenerative mechanisms, leading to functional impairments in sensory, motor, and autonomic pathways [2,3]. The primary etiological factors for nerve lesions include traumatic injuries, such as penetrating wounds, blunt force trauma, traction, and crush injuries [4]. Nerve damage may also arise from compressive neuropathies caused by prolonged external pressure, tumors, hematomas, or anatomical entrapments; ischemic mechanisms, including vascular compromise or systemic conditions such as diabetes mellitus; and inflammatory or autoimmune disorders, such as Guillain–Barré syndrome or chronic inflammatory demyelinating polyneuropathy [5].

Based on the mechanism of injury, peripheral nerve injuries are classified into open and closed injuries. Open injuries are caused by penetrating trauma, including lacerations, gunshot wounds, and surgical interventions, leading to partial or complete nerve transection. These injuries often require direct microsurgical repair or nerve grafting for functional restoration. In contrast, closed injuries occur without external wounds and result from blunt trauma, excessive traction, compression, or ischemia. Examples include nerve contusions from fractures or dislocations, stretch-induced nerve damage, and entrapment neuropathies. The classification of peripheral nerve injuries is critical for determining the appropriate therapeutic strategy and predicting functional recovery [2,6,7,8,9].

Peripheral nerve injuries vary in mechanism and severity, influencing the complexity of their treatment and recovery. Although peripheral nerves possess an intrinsic regenerative capacity, the healing process remains highly unpredictable and is largely dependent on the extent and nature of the injury, the patient’s age, and the timing of treatment. As a result, achieving full sensory and functional recovery remains a significant clinical challenge. This has driven extensive research efforts aimed at elucidating the mechanisms of nerve injury, repair, and regeneration [1,2,10].

The introduction of the operating microscope, advancements in microsurgical techniques, improved imaging technologies, and a deeper understanding of peripheral nerve microanatomy have significantly enhanced functional outcomes in nerve repair. Additionally, progress in both basic science and clinical research has provided valuable insights into the pathophysiology of nerve injury, regeneration, and repair mechanisms, further refining treatment strategies and improving patient prognosis [8,11].

Despite these innovations, achieving complete sensory and motor recovery remains a significant challenge, driving the ongoing research in peripheral nerve reconstruction.

In this narrative review, we aim to provide a detailed overview of peripheral nerve injuries, both from a pathophysiological perspective and in terms of subsequent therapeutic guidance. Additionally, we will review existing reconstructive options and emerging promising strategies designed to improve functional outcomes.

### 1.2. Classification of Peripheral Nerve Injuries

Various classification systems were introduced to describe the severity of peripheral nerve injuries, the most common being the Seddon and Sunderland Classifications (presented in Table 1):

The Seddon classification is simpler and more clinically oriented, making it easier to use in daily practice. However, it lacks the detailed gradation of nerve injury severity provided by the Sunderland classification. The Sunderland classification offers a more precise assessment of nerve damage, which can be useful in research and complex clinical cases [7,13,14].

The Seddon and Sunderland classifications are essential for determining the prognosis and guiding the treatment of peripheral nerve injuries. For example, neurapraxia (first-degree Sunderland) typically resolves spontaneously within a few months, while neurotmesis (fifth-degree Sunderland) often requires surgical intervention for any meaningful recovery [7,13,14,15].

Peripheral nerve injuries are complex conditions that greatly affect patients’ quality of life and functional recovery. The classifications proposed by Seddon and Sunderland remain the standard for assessing nerve injuries, providing a fundamental understanding of injury severity and guiding treatment. However, these traditional systems have certain limitations in fully capturing the complexity of injury characteristics, treatment strategies, and prognostic factors. Advances in medical research have led to the development of newer classifications that aim to complement and refine these existing models.

A novel classification system for peripheral nerve injuries has been introduced by Lavorato et al. following a retrospective analysis of 24 patients with traumatic nerve damage. This system integrates both injury-specific factors and prognostic codes, offering a more comprehensive and clinically relevant framework for assessing peripheral nerve injuries. Unlike traditional models such as Seddon’s and Sunderland’s classifications, which primarily categorize injuries based on structural damage, this new system accounts for additional variables such as the specific nerve involved, the anatomical site of the lesion, the type of nerve function affected (motor, sensory, or mixed), and the extent of surrounding tissue involvement. Moreover, it introduces prognostic codes that incorporate key determinants of functional recovery, including patient age, timing of intervention, surgical approach feasibility, and relevant comorbidities. By encapsulating these parameters into an alphanumeric coding system, this classification enhances clinical decision-making by providing a standardized method for describing injury severity, guiding treatment strategies, and predicting outcomes. The inclusion of prognostic elements marks a significant improvement over existing systems, facilitating early intervention planning and interdisciplinary communication. Furthermore, its structured approach allows for more precise comparisons of treatment efficacy across studies, thereby contributing to evidence-based advancements in peripheral nerve repair and rehabilitation [16].

An electrophysiology-based classification system for peripheral nerve injuries has been proposed to enhance diagnostic accuracy and prognostication by incorporating objective, quantifiable data from nerve conduction studies (NCS) and electromyography (EMG) [17,18]. This classification refines traditional models by distinguishing between different grades of nerve injury based on electrophysiological findings. For instance, neurapraxia (Grade 1) is characterized by normal EMG and NCS distal to the injury site, with a conduction block proximally, indicating transient functional impairment without axonal disruption. In contrast, axonotmesis (Grade 2) presents with reduced or absent compound muscle action potentials (CMAPs) and sensory nerve action potentials (SNAPs), reflecting axonal injury with preserved connective tissue structures. Neurotmesis (Grade 5), the most severe form, is marked by a complete loss of CMAPs and SNAPs, signifying total nerve disruption. This electrophysiological approach improves upon traditional classification systems by providing early, precise, and reproducible measurements of nerve function, allowing for a more accurate diagnosis and prognosis. Additionally, it enables clinicians to monitor nerve recovery over time, informing rehabilitation strategies and optimizing intervention timing. The integration of electrophysiology into peripheral nerve injury classification has the potential to standardize assessment, reduce diagnostic ambiguity, and improve treatment outcomes by guiding surgical decision-making and patient management [17,18].

### 1.3. Physiopathology of Peripheral Nerve Lesions

**Figure 1:**
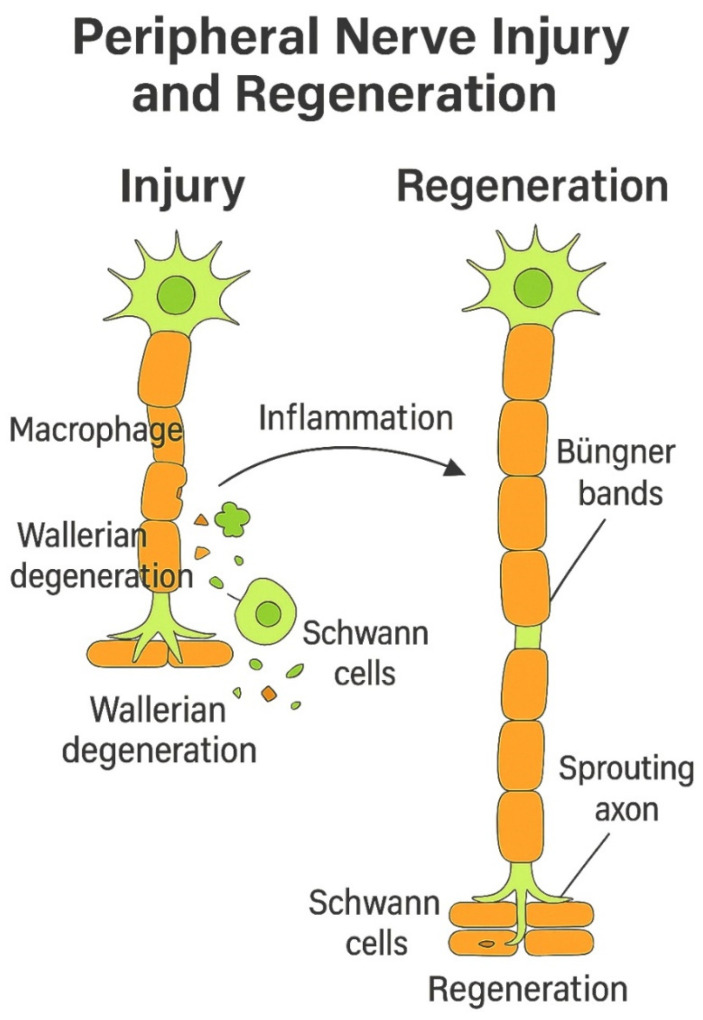
Wallerian degeneration and axonal regeneration following peripheral nerve injury.

Peripheral nerve injury initiates a series of pathophysiological responses, including neural tissue edema and ischemia, as well as disrupted axonal transport. The subsequent repair and regeneration process is highly complex, involving inflammation, Wallerian degeneration, neovascularization, Schwann cell activation, proliferation, migration, Büngner bands (endoneurial pathways), and neurite extension (Figure 1) [19,20].

Proximally to the site of injury, axons retract to a certain extent and enter a temporary inactive phase. During this period, molecular signals triggered by the injury begin to take effect, and neurotrophic factors are transported to prepare for the development of a regenerating structure with a single elongating axon branching into multiple smaller axons. In myelinated nerves, these sprouting axons extend through natural gaps in the myelin sheath, known as the nodes of Ranvier, and advance toward their intended sensory or motor destinations [21,22,23].

Nerve injury triggers significant changes in axons, Schwann cells, macrophages, and fibroblasts, supporting the theory that different components of a neuron respond independently to trauma. Proximal injuries, such as avulsions, result in soma loss and neuronal death, while distal damage preserves the soma and its regenerative capacity. Wallerian degeneration, first described by Waller in 1850, occurs after axonal severance, leading to cellular disintegration, disrupted intracellular transport, and the loss of electrical signaling, all phenomena occurring distally to the injury site. Notably, neuromuscular synapses degrade hours before Wallerian degeneration, indicating an independent process [24,25,26].

Schwann cells remain intact but undergo phenotypic changes, while macrophages and endothelial modifications aid in debris clearance and the removal of other inhibitors that could prevent axonal regeneration. Thus, macrophages rapidly migrate to the affected site, responding to microenvironmental changes and secreting factors like VEGF-A to mitigate hypoxia, which, in turn, modulates Schwann cell function. Simultaneously, distal Schwann cells, upon losing contact with proximal axons, undergo a dedifferentiation process, phagocytizing myelin and axonal debris while releasing cytokines to recruit additional macrophages [27,28,29].

Recent studies highlight the role of Toll-like receptors (TLRs) in Schwann cell activation. TLRs recognize damage signals and promote monocyte chemotactic protein-1 (MCP-1) expression. In vivo studies showed reduced IL-1β and MCP-1 levels in injured sciatic nerves of TLR-deficient mice, impairing macrophage recruitment and slowing degeneration. However, the partial reduction suggests additional pathways contribute to the regenerative response. Inflammatory cytokines such as TNF-α, IL-1α, and IL-1β further regulate immune cell recruitment, sustaining the healing process. Activated macrophages amplify this response by secreting the same cytokines, perpetuating nerve regeneration [30,31,32].

Schwann cells are the primary mediators of nerve regeneration in the peripheral nervous system, unlike CNS glial cells, which contribute to scarring. Following nerve injury, Schwann cells dedifferentiate into a migratory, proliferative, but nonmyelinating phenotype marked by p75 neurotrophin receptor (p75NTR), L1, and NCAM. Their maturation is regulated by ERK, p38 MAPK, and AKT signaling, with NF-κβ playing a key role in myelination and neuregulin1 (NRG1) type III, further influencing axonal ensheathment and Schwann cell differentiation [33].

Migration, while mainly studied in development, involves the Trk activation of Rho GTPases, ErbB–NRG interactions, and extracellular matrix components such as laminins and integrins. Schwann cells also interact with fibroblasts via ephrin-B/EphB2 signaling to ensure organized regeneration. Excess Schwann cells that lack axonal contact undergo apoptosis, likely mediated by NF-κβ inhibition. Advanced imaging techniques, such as Schwann cells labeled by green fluorescent protein in transgenic mice, have demonstrated that Schwann cell migration precedes axonal sprouting in acellular grafts and conduits, with more prolific proliferation distally, offering new insights into peripheral nerve regeneration [34,35,36].

Schwann cells located distal to the injury site are crucial for axon regrowth, as they undergo dedifferentiation and organize into longitudinal cell strands known as bands of Büngner. These structures, consisting of hundreds of pro-regenerative microchannels, serve as aligned tubular guidance pathways that direct axonal regeneration. If regenerating axons stray from the bands of Büngner, their elongation halts, leading to the formation of a painful neuroma [37,38,39].

After nerve injury, prolonged regeneration times contribute to poor functional recovery due to muscle atrophy and fibrosis. While short-term denervated muscles can recover well, chronic denervation leads to irreversible damage. Additionally, Schwann cells in the distal nerve stump decline in number and downregulate trophic factor production, reducing their support for regenerating axons. This results in fewer axons reaching their targets, significantly impairing functional recovery [40,41,42].

Laminins, integrins, and dystroglycans play a crucial role in nerve regeneration by regulating the Schwann cell phenotype, providing guidance for regenerating axons, and supporting myelination. Laminins 2 and 8 are upregulated following nerve injury, and their absence impairs axonal regeneration, as seen in knockout studies. Integrins mediate laminin signaling, which promotes cytoskeletal elongation and axonal growth. Laminins also regulate Schwann cell–axon interactions, with β1 integrin facilitating axonal sorting and dystroglycan aiding myelin folding. Additionally, fibrin, though used in nerve repair, inhibits myelination by keeping Schwann cells in a dedifferentiated state until the plasminogen activator clears it, allowing remyelination. These extracellular matrix components highlight the superiority of autografts, which contain both Schwann cells and laminin, over acellular allografts and empty conduits, significantly influencing nerve regeneration outcomes [43,44,45].

Neurotrophism refers to the process by which neurotrophic factors, either autocrine or paracrine, support axonal growth and development. Successful recovery depends on the number of regenerating motor and sensory axons that successfully reconnect with their respective targets, such as motor end plates or sensory receptors. In addition, nerve regeneration exhibits neurotropism, which is an inherent ability of axons to grow toward their specific end-organ targets, where they receive crucial survival signals that help prevent neuronal apoptosis. The significance of neurotropism in nerve repair has been demonstrated in cases where end-organ connections are disrupted after nerve injury and subsequent repair, leading to dramatically reduced axonal regrowth. A clear distinction between neurotrophism and neurotropism can be seen when genetic modifications cause nerve cells to continuously overproduce the glial-cell-line-derived neurotrophic factor (GDNF), which is normally only temporarily present in the injured distal segment and denervated muscle. The persistent overexpression of this factor leads to an overwhelming neurotropic effect, resulting in axons becoming trapped and failing to properly reconnect with their target organs [46,47,48,49,50].

Neurotrophins and neurotrophic factors regulate axonal regeneration and Schwann cell behavior through specific receptor interactions. Neurotrophins, including NGF, BDNF, NT3, and NT4/5, bind Trk receptors and p75NTR, influencing neuronal survival and myelination. GDNF and its related ligands (NRTN, ARTN, and PSPN) signal through the Ret tyrosine kinase and GFRα coreceptors, playing a crucial role in motor neuron survival and regeneration. Following nerve injury, GDNF expression is upregulated in the distal nerve stump and muscle, while its receptors show differential regulation based on the injury location and developmental stage. GDNF delivery enhances motor axon regeneration, with its effects being location-dependent and more pronounced in chronic nerve injury. Research suggests that GDNF-mediated signaling supports motoneuron regeneration by forming trophic gradients that guide axonal growth, offering potential therapeutic applications in peripheral nerve repair [51,52,53,54,55].

## 2. Methodology

This narrative review summarizes the current evidence on surgical techniques, biomolecular interventions, and regenerative strategies for optimizing peripheral nerve regeneration. A comprehensive literature search was performed using academic databases including PubMed, Google Scholar, and Web of Science, covering relevant publications in the field published up to the year 2025. Key search terms included “peripheral nerve injury”, “nerve regeneration”, “nerve repair”, “nerve graft”, “nerve conduits”, “growth factors”, “stem cells”, “gene therapy”, “nanotechnology”, and “electrical stimulation”.

Relevant articles were identified based on their titles and abstracts, followed by a detailed full-text review. Inclusion criteria comprised original research studies, clinical trials, animal studies, and reviews published in the English language. We excluded studies exclusively addressing central nervous system regeneration or conditions unrelated to peripheral nerve injury.

The selected literature was thoroughly assessed and synthesized to reveal significant findings, current therapeutic options, and emerging trends, providing clinicians and researchers with an updated overview of peripheral nerve regeneration strategies.

## 3. Discussion and Literature Review on Therapeutic Management of Peripheral Nerve Injuries

When the components of a peripheral nerve are completely disrupted or the nerve is transected, the only therapeutic approach is surgical treatment. The choice of repair strategy depends on the severity of the injury, the size of the nerve gap, and the available resources. The goal of a high-quality nerve repair is to correctly guide the regenerating fibers toward the appropriate environment of the distal end, minimizing the loss of regenerative units at the suture line or along incorrect regenerative pathways [3].

### 3.1. Direct Nerve Repair

For clean transections with minimal nerve gaps, direct neurorrhaphy is the preferred approach, representing the gold standard for treating peripheral nerve injuries in environments with adequate vascularization and well-perfused soft tissue, ensuring optimal conditions for healing and regeneration. The primary end-to-end nerve repair, the most used technique, consists of four main steps: preparation, approximation, alignment, and maintenance [1,56,57].

The process begins with the preparation of the nerve ends, which often requires resections or interfascicular dissections to separate individual fascicles or groups of fascicles. The preparation of the nerve ends involves the careful excision of necrotic or non-viable tissue using surgical blades, ensuring that only healthy, well-vascularized tissue remains for coaptation. If additional length is needed, joint flexion or bone shortening may be considered. Microscopic dissection is used to prepare the nerve ends and ensure a clean repair site with optimal conditions for healing [56].

Next, the nerve ends are approximated, with careful attention to the gap between them and the level of tension to prevent complications. Studies show that tensionless repairs lead to better outcomes. While some mobilization of the nerve ends is necessary, extensive interfascicular dissection should be avoided to prevent additional damage [9,56,58].

Once positioned, the nerve ends are coapted to ensure optimal alignment and contact between fascicles. Alignment is a critical step, requiring the proper positioning of the nerve ends. Blood vessels must be aligned correctly, and rotational alignment must be carefully maintained to ensure functional recovery. Direct neurorrhaphy connects the severed ends precisely, either fascicle to fascicle or group to group, while indirect coaptation achieves the same outcome using a nerve graft or a nerve conduit [56,59].

To complete the repair, epineural sutures are placed using fine 9-0 or 10-0 non-absorbable threads, ensuring a stable approximation of the nerve ends and supporting the healing process. These sutures hold the repair in place and prevent malrotation. In some cases, particularly with larger nerves, individual fascicular groups may be identified and sutured separately (fascicular group nerve repair) to improve sensory and motor function recovery [56,60].

When performed promptly after the injury, the tension-free microsurgical suturing of the nerve ends using epineural or perineural sutures under magnification, preferably using the operative microscope, offers the highest potential for recovery by preserving the anatomical continuity of the nerve [61,62].

**Figure 2:**
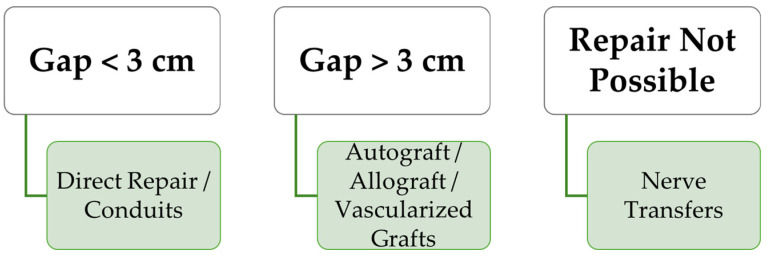
Treatment options for peripheral nerve injuries based on nerve gap size.

When a nerve cannot be repaired by direct neurorrhaphy, there are some options that a surgeon must consider, each with its indications and advantages: autologous nerve grafting, vascularized nerve grafting, nerve conduits, allografts, and nerve transfers (Figure 2) [8,56,61,62]. In cases where a nerve gap prevents direct repair, nerve grafting is the standard solution.

### 3.2. Autologous Nerve Grafts

Autografts, harvested from the patient’s own body, are considered the gold standard due to their excellent biocompatibility and structural support for axonal regeneration.

The first nerve graft was performed by Phillipeaux and Vulpian in 1870. In 1939, Bunnel and Boyes documented their experience with thin autogenous nerve grafts, reporting promising results. Shortly after, the clinical success of free autologous nerve grafting improved with the introduction of cable grafts, which enhanced graft revascularization and helped prevent central necrosis in larger grafts [63,64,65].

Autografts are indicated in bridging nerve grafts longer than 3 cm, more proximal injuries, and critical nerve injuries [3]. This technique involves harvesting functionally less important nerves from different donor sites, including the sural nerve, superficial cutaneous nerves, or the lateral femoral cutaneous nerve. The selection of the most appropriate donor nerve requires the careful consideration of several factors, including the size of the nerve gap, the anatomical location of the nerve repair, and potential donor site morbidity. A well-matched graft should provide adequate structural support while minimizing functional loss at the harvest site [3,9,66].

Despite its advantages, the use of autologous nerve grafts has several limitations. Donor site morbidity is a significant concern, as harvesting a nerve graft involves an additional surgical procedure, which increases the risk of postoperative complications such as scarring, painful neuroma formation, and localized sensory deficits. The availability of suitable graft tissue is limited, presenting a significant challenge in cases of extensive nerve defects that necessitate long or multiple grafts. This limitation may compromise the ability to achieve adequate nerve reconstruction and functional recovery, especially when the donor nerve length or caliber is insufficient for optimal repair [3,9,62,66,67,68].

Moreover, autografting requires a secondary incision for graft procurement, further contributing to patient discomfort and prolonging recovery. Another challenge is fascicular mismatch, where the structural differences between the donor and recipient nerves may impair optimal regeneration, potentially affecting functional outcomes [3,69].

Non-vascularized nerve grafts are transferred without a vascular supply and require the presence of a recipient bed for revascularization. An increased vascular supply is necessary for Schwann cell survival and to prevent intraneural fibrosis. For this reason, non-vascularized nerve grafts are indicated only in cases where the recipient bed allows for rapid revascularization. For large nerve gaps or cases where the vascular supply is compromised, vascularized nerve grafts are employed. These grafts, which include their blood supply, enhance the survival and regenerative potential of the transplanted nerve tissue [65,70,71,72].

The first reported vascularized nerve graft in the upper extremity was described by Strange in 1947, transferring the ulnar nerve as a pedicled flap to restore the continuity of the median nerve in cases where both nerves have significant defects. In 1976, Taylor and Ham performed the first free vascularized nerve graft, using a 24 cm segment of the superficial radial nerve, supplied by the radial artery, in a free tissue transfer manner, to reconstruct the median nerve in a patient with Volkmann’s ischemic contracture. Since then, numerous experimental and clinical studies have investigated the effectiveness of vascularized nerve grafts to establish their specific indications [65,71,73,74].

Vascularized nerve grafts should be considered in cases where non-vascularized nerve grafts may be insufficient, such as nerve gaps longer than 6 cm, poor vascularized recipient beds, and composite defects requiring a free flap. They are also indicated for proximal nerve injuries, including brachial plexus lesions, as well as in cases of delayed reinnervation, where faster nerve regeneration may help prevent muscle atrophy. The availability of a pedicled donor nerve within the surgical field—for instance, the great auricular nerve during facial nerve reconstruction in parotidectomy cases—represents another clinical indication. Vascularized grafts can be beneficial when radiation therapy is planned, as it may delay revascularization. Additionally, older patients may experience improved nerve regeneration with vascularized nerve grafts [65,71].

While non-vascularized nerve grafts remain the standard for most nerve reconstructions, vascularized grafts, though more technically demanding, provide significant advantages in complex cases where improved vascularization is essential for graft survival and optimal functional recovery [70,71].

### 3.3. Nerve Transfers

In situations where neither direct repair nor grafting is feasible, nerve transfers are considered. This approach involves re-routing intact regional nerves to restore function to denervated areas. While effective, nerve transfers often require sacrificing the donor nerve’s original function [75,76].

There are no strict protocols for determining when to perform nerve transfers, but there is a general set of criteria to customize each patient’s procedure. These indications include proximal injuries (such as, but not limited to, brachial plexus injury), significant distance from the target motor end plates, late presentation after the injury, and extensive limb trauma leading to segmental nerve function loss, as well as prior injury resulting in substantial scarring around essential skeletal or vascular structures. Nerve transfers offer a degree of reassurance for functional recovery in cases where nerve grafts or primary repair may be unreliable. However, in more complex injuries, even more innovative reconstructive approaches may be necessary [8,76].

For all nerve transfers, selecting an appropriate donor nerve requires considering several key factors: the donor nerve’s proximity to the recipient nerve’s motor end plates, the availability of a redundant or expendable donor, the synergistic function between donor and target muscles, the similarity in the number of motor or sensory axons, and proper size matching [8,77].

For example, distal radial nerve function can be restored following proximal injury using redundant motor branches from the median nerve, such as branches for the flexor carpi radialis or flexor digitorum superficialis muscles. On the other hand, a proximal ulnar nerve injury presents a significant challenge, as even timely repair often yields only protective sensation in the digits without meaningful recovery of intrinsic hand muscle function. To address this, a distal nerve transfer of the terminal anterior interosseous nerve (the branch to the pronator quadratus muscle) to the distal ulnar motor fascicle can facilitate reinnervation before neuromuscular junction degeneration occurs. Median nerve transfers, depending on the location, may involve either the distal anterior interosseous nerve to the motor recurrent branch for the thenar muscles to restore thumb opposition, or branches to flexor carpi ulnaris transferred to the anterior interosseous nerve to restore finger flexion. According to the need of reinnervation, several other nerve transfers have been described [8,77,78,79,80,81].

### 3.4. End-to-Side Coaptation

Originally described more than 100 years ago, end-to-side coaptation serves as an alternative when the proximal nerve stump is unavailable or inaccessible. Interest in this method was reignited in 1994 when Viterbo et al. demonstrated axonal regeneration using end-to-side neurorrhaphy in a rat model. In this technique, the injured distal stump is connected to the side of an intact donor nerve. While this technique has many supporters, some researchers have reported less promising outcomes, with no evidence of reinnervation through end-to-side coaptation [8,82,83].

In end-to-side repair, the term “collateral sprouting” describes the process of new axonal growth from the donor nerve into the recipient stump. However, the exact source of these axons remains a subject of debate. In rat models in which the donor nerve remained intact, evidence of collateral sprouting was observed, even without donor nerve injury, but further research in more advanced models showed that only sensory axons exhibited de novo sprouting without injury. Moreover, other animal models proved that donor nerve injury is necessary for motor regeneration following end-to-side repair [8,84,85,86].

Based on this evidence, excellent outcomes can be achieved in clinical practice with minimal morbidity through carefully planned nerve transfers restricted to reconstructing noncritical sensory deficits [8,83].

### 3.5. Nerve Allograft Transplantation

Nerve allograft transplantation is a valuable option for repairing severe peripheral nerve injuries that cannot be addressed through conventional methods like autologous nerve grafting or nerve transfer. However, the necessity for systemic immunosuppression, even if temporary for nerve allograft recipients, limited its broader clinical application [87].

Peripheral nerve allografts are a type of tissue allograft that can be transplanted as either vascularized or non-vascularized grafts. Vascularized peripheral nerve allografts are generally used for larger diameter or longer nerve segments, such as in hand transplantation, while non-vascularized peripheral nerve allografts are typically employed for smaller diameters or shorter segments, which may not undergo immediate revascularization [88,89,90].

Allografts, derived from deceased donors, provide an alternative that eliminates donor site morbidity and offers better size matching [8,91].

With the advent of modern immunosuppressants, the improved understanding of nerve alloimmunity, and precise microsurgical techniques, isolated nerve allografts have become clinically feasible with limited immunosuppression [87].

Schwann cells, through ICAM-1 and MHC II expression, act as antigen-presenting cells, triggering a rapid immune response after transplantation. As a result, the graft undergoes a state of chimerism and is progressively replaced by host tissue. This phenomenon underscores the need for finite host immunosuppression. Due to the eventual replacement of Schwann cells by the recipient, the nerves are accepted after an initial immune response, leading to a reduced cellular infiltrate. Additionally, studies have shown that nerves induce a low Th1 cytokine profile, further reducing their antigenicity. Evidence from hand transplantation studies supports this low antigenicity, as biopsies from clinically rejected nerve allografts show perineural inflammation but no significant neuritis, even in severe rejection cases [8,92].

The low immunogenicity of peripheral nerves allows for the administration of reduced doses of limited immunosuppression, facilitating nerve defect reconstruction in cases where autologous nerve repair is not possible [87,92].

Despite the risks associated with systemic immunosuppression, nerve allografting remains a field of great interest. Various strategies, including cold preservation, irradiation, and lyophilization, have been explored to reduce nerve allograft antigenicity [8,93].

Ray and Mackinnon proposed a clinical protocol for nerve allografting which include ABO-matched donors, small-diameter donor nerves, cold preservation at 4–5 °C for seven days, three days of pretreatment with FK506, and continued immunosuppression until Tinel’s sign progresses beyond the distal graft site [8]. Unlike solid organ transplantation, nerve allografts require only temporary immunosuppression. Once sufficient migration of host Schwann cells occurs—typically within 24 months—systemic immunosuppression can be discontinued. Additionally, despite its associated risks, FK-506 (tacrolimus), a commonly used immunosuppressant, has been shown to enhance nerve regeneration. Following systemic administration of tacrolimus in upper limb transplantation, remarkable rates of nerve regeneration has been observed, with rapid Tinel sign progression and early reinnervation of intrinsic hand muscles [8,94,95].

Current research focuses on understanding the host immune response to nerve allotransplantation and identifying ways to modify alloantigen recognition and presentation. Advancing knowledge in this field could refine immunomodulation strategies and possibly provide an unlimited supply of nerve graft material. The current objective is to minimize immunosuppression to a level that sustains allograft function while preventing adverse reactions. Ongoing research is focused on developing novel immunosuppressive therapies to avoid toxicity and promote donor-specific tolerance. Combining the costimulatory blockade—and, therefore, interfering with T-cell activation—with other immunomodulatory approaches, such as cold preservation, which influence both indirect and direct pathways, may offer a viable alternative to systemic immunosuppression after nerve allografting. However, careful patient selection and the cautious application of these techniques remain essential [8,96,97,98].

### 3.6. Nerve Conduits

Nerve conduits represent a promising alternative to nerve grafts, typically used for nerve gaps smaller than 3 cm. They serve as scaffolds that facilitate axonal regeneration while preventing the protrusion of endoneural contents during the healing process. These tubular structures can be made from synthetic or biological materials. The ideal nerve conduit should be biocompatible, ensuring a non-toxic environment while minimizing immunological reactions and inflammation. It should also be biodegradable to prevent nerve compression and sufficiently permeable to allow the proper exchange of oxygen and nutrients, thereby creating a microenvironment conducive to nerve regeneration. Another important property of nerve conduits is flexibility. An ideal nerve conduit should be flexible enough to adjust itself to different planes without producing lesions to growing axons and nearby structures, but not too flexible so that it causes the collapse or rupture of the conduit [61,99,100,101,102,103,104,105]. Table 2 presents a classification of nerve conduits, along with a description of their properties and indications [31,61,106,107,108,109,110,111,112,113].

#### 3.6.1. Autograft-Based Conduits

In addition to autologous nerve grafts, which have been widely used as the gold standard for bridging nerve gaps, other autologous tissues can also be utilized to create nerve conduits. These conduits can be derived from blood vessels, such as veins or arteries, fascia, fibers from adjacent muscles, bone, etc. The advantages of using autologous nerve conduits over nerve grafts include greater availability, the ability to remain within the same surgical field, and limited donor site morbidity. However, the results tend to be inferior compared to nerve grafts, particularly in defects larger than 1–2 cm [62,104,114,115,116,117].

#### 3.6.2. Synthetic Nerve Conduits

Synthetic nerve conduits were developed to address the need for bridging nerve gaps without the drawbacks associated with harvesting autologous nerve grafts. A wide variety of materials can be used to fabricate these conduits, including both natural and synthetic polymers. Natural polymers may be derived from proteins (such as gelatin, laminin, collagen, and silk) or polysaccharides (such as chitosan and cellulose). Synthetic polymers can be either permanent, like silicone, or biodegradable, such as polylactic acid, polyglycolic acid, and polycaprolactone. The advantage of synthetic polymers over natural ones lies in their superior mechanical support [69,111,118,119,120].

##### Natural Polymers

Collagen is widely used due to its biocompatibility and ability to support cell adhesion and proliferation. For instance, collagen-sponge-filled conduits have been shown to promote superior nerve regeneration compared to collagen fibers [121,122]. Additionally, collagen filaments have been successfully used to bridge 30 mm nerve defects in rat models [122].

A protein derived from silk, sericin, has been used in nerve guidance conduits. Sericin-based conduits have been shown to promote Schwann cell proliferation and upregulate neurotrophic factors, leading to a functional recovery comparable to autografts [123].

Silk-based conduits have gained attention for their biocompatibility and mechanical strength. Silk nanofibers with hierarchical anisotropic architectures have been developed to mimic the natural nerve structure, supporting Schwann cell proliferation and axonal growth [124,125].

##### Synthetic Polymers

Polyglycolic acid (PGA) is a biodegradable polymer often used in combination with other materials. A chitosan/PGA conduit has been shown to successfully bridge a 30 mm nerve defect in a dog model, restoring nerve continuity and functional recovery [126].

Polylactic-co-caprolactone (PCL) is a biodegradable polymer with excellent mechanical properties. Electrospun PCL/collagen nanofibers have been used in conductive nerve guidance conduits, promoting Schwann cell elongation and neurite outgrowth [127].

Polycaprolactone-based scaffolds have been combined with graphene and polypyrrole to create conductive 3D scaffolds for nerve regeneration. These scaffolds have shown non-cytotoxicity and the ability to replicate the properties of native tissue [128].

##### Composite Materials

Composite materials combine natural and synthetic polymers to leverage their respective advantages. Chitosan/PGA has been used to create dual-component artificial nerve grafts, which have been successfully tested in dog models [126]. Silk fibroin conduits loaded with PEDOT nanoparticles have been developed for their electroconductive properties. These conduits have shown excellent mechanical and biological performance by preventing scar tissue formation and promoting Schwann cell growth [129]. PCL/Gelatin/Polypyrrole/Graphene is a composite scaffold that combines the flexibility of PCL with the conductivity of polypyrrole and graphene, creating a conductive environment for nerve regeneration [128].

##### Ceramics and Other Materials

Silica fibers have been incorporated into nerve conduits for their biocompatibility and structural support. These fibers are fabricated using sol–gel electrospinning techniques [130]. Hyaluronic acid (HA)-based hydrogels have been used in injectable nerve conduits. A self-healing HA-based hydrogel has been shown to promote nerve regeneration by activating the IL-17 signaling pathway and enhancing Schwann cell myelination [131].

## 4. Emerging Trends and Future Directions in Nerve Regeneration

**Figure 3:**
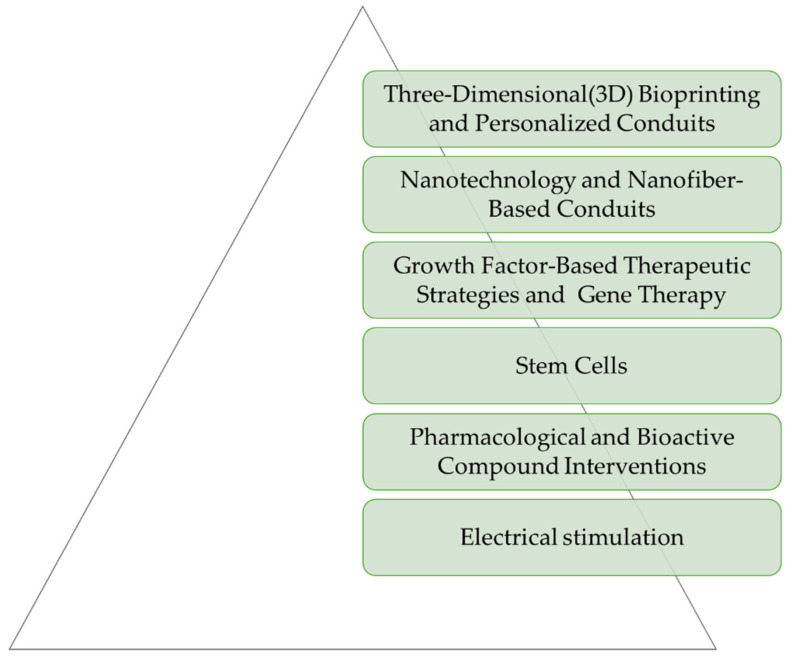
Emerging trends and future directions in nerve regeneration.

While peripheral nerves possess an intrinsic regenerative capacity, severe injuries require intervention for functional recovery. Current treatment options, such as surgical repair and nerve grafts, are limited by complications like poor axonal regrowth and misdirected reinnervation. The field of nerve regeneration continues to evolve, with emerging trends and future directions in the development of nerve conduits being focused on improving their performance and expanding their clinical applications. Researchers are exploring novel materials, advanced manufacturing techniquesm and the integration of new biological and technological advancements to improve nerve repair outcomes, enhancing nerve regeneration, as depicted in Figure 3 [132,133].

### 4.1. Three-Dimensional (3D) Bioprinting and Personalized Conduits

Three-dimensional printing arose as an innovative technique for producing nerve conduits. It allows the fabrication of conduits from various materials, constructing structures layer by layer. This approach enables the creation of custom combinations of materials and cells essential for promoting nerve regeneration, while also ensuring adequate support and biocompatibility. Another advantage of 3D printing is the ability to craft conduits specifically designed to fit individual patients, taking into consideration the injured nerve, the site of lesion, and nerve anatomy based on imaging (MRI or CT scans) [134,135,136].

We found that 3D printing allows for the creation of nerve conduits that are tailored to the specific anatomical and injury-related needs of the patient. This is achieved by using imaging data to design conduits that precisely fit the nerve defect, ensuring optimal support and integration [134].

Advanced 3D-printing techniques allow for the fabrication of conduits with complex geometries, such as multichannel or bifurcating designs, which can better mimic the natural architecture of nerves and enhance regeneration outcomes [137].

Bio 3D conduits are often made from human umbilical cord-derived mesenchymal stromal cells (UC-MSCs), which have shown superior nerve regeneration capabilities compared to traditional materials like silicone tubes. In a rat model, Bio 3D conduits demonstrated better outcomes in terms of axon diameter and myelination, with reduced rejection rates compared to allografts [138].

The use of multifunctional electroactive bioinks in 3D bioprinting, such as those based on gelatin methacrylate (GelMA) combined with conductive materials like carbon nanofibers, enhances the mechanical and electrical properties of the conduits. These materials support high neural cell viability and can facilitate the functional regeneration of nerve tissues [139].

In addition, 3D printing can combine nerve conduits with materials like poly (glycerol sebacate) acrylate (PGSA) composites, which incorporate conductive elements such as silver nanoparticles and graphene, offer high biodegradability, and promote cell proliferation, ensuring the long-term success of nerve regeneration [140].

### 4.2. Nanotechnology- and Nanofiber-Based Conduits

Nanotechnology- and nanofiber-based conduits represent a pivotal advancement in regenerative medicine, particularly in the development of nerve conduits designed to replicate the intricate nanostructure of native nerve matrices. Among these, materials such as graphene and carbon nanotubes have garnered significant attention due to their exceptional mechanical properties, electrical conductivity, and flexibility—attributes that are crucial for facilitating nerve regeneration. Graphene has been extensively studied for its potential to promote axonal regrowth and enhance neural regeneration. This growing interest in graphene-based and nanocomposite conduits stems from their potential to provide an optimized microenvironment for nerve repair. However, despite their promise, challenges related to biocompatibility, cytotoxicity, and large-scale fabrication remain key barriers to clinical translation [141,142,143].

The structural and functional advantages of graphene and carbon nanotubes have positioned them as promising candidates for nerve conduit applications. Graphene is a material that is based on carbon atoms organized in a single layer. This structure provides graphene with exceptional electrical properties, strength, and flexibility. Carbon-based materials are largely used in neuroscience due to their properties. The widespread use of graphene in the central nervous system has been observed to enhance axonal regrowth and support nerve regeneration. This finding sparked a growing interest in the use of graphene as a material for nerve conduits [141,144,145,146,147].

Graphene’s single-layer carbon atom structure imparts exceptional electrical properties, resistance, and flexibility, making it an ideal substrate for nerve conduits [148,149].

Carbon nanotubes, when incorporated into composite fibers, significantly enhance the mechanical strength and elasticity of nerve conduits, as demonstrated by increased tensile strength and improved elastic characteristics in experimental studies [150].

Furthermore, graphene-integrated conduits have been shown to actively promote axonal regrowth and nerve regeneration, underscoring their potential in peripheral nerve injury repair [149].

Beyond material properties and fabrication techniques, the biological outcomes of graphene-based conduits further reinforce their therapeutic potential. Preclinical studies have demonstrated that these conduits significantly improve nerve function recovery, as evidenced by enhanced compound muscle action potential (CMAP) and increased nerve conduction velocity (NCV) in animal models. Moreover, incorporating dual neurotrophins into nanofibrous scaffolds has been shown to facilitate Schwann cell proliferation and differentiation, further amplifying nerve regeneration capacity [151].

Multifunctional conduits, such as those embedded with nanosilver, have additionally demonstrated antimicrobial properties while simultaneously supporting neural tissue repair, which is particularly beneficial in contaminated environments [152].

### 4.3. Growth Factor-Based Therapeutic Strategies and the Role of Gene Therapy for Peripheral Nerve Regeneration

Growth factors (GFs) have emerged as a promising strategy for enhancing nerve regeneration by promoting cellular survival, axonal outgrowth, and remyelination [132].

GFs are essential polypeptides that regulate neural survival, differentiation, and axonal growth. In response to nerve injury, Schwann cells and neurons secrete neurotrophic factors such as nerve growth factor (NGF), brain-derived neurotrophic factor (BDNF), fibroblast growth factors (FGFs), and glial-cell-line-derived neurotrophic factor (GDNF) to modulate the regenerative microenvironment. However, endogenous GF levels are often insufficient for optimal nerve repair, necessitating exogenous supplementation. Studies have demonstrated that GF administration can improve axonal regeneration, remyelination, and functional recovery [132,153].

One of the main problems in growth factor therapy is their rapid degradation and limited half-life in biological fluids, which significantly reduces their therapeutic efficacy. To address this, biomaterial-based delivery systems have been developed to provide a sustained and localized release of GFs at the injury site. Nerve conduits made from biodegradable or nondegradable materials serve as scaffolds that support nerve regeneration while preventing scar tissue formation. Another important drawback of neurotrophic factors is that the result is dose-dependent. Fortunately, there are interesting avenues focusing on the delivery of these factors. The incorporation of GFs into these conduits through controlled-release mechanisms, such as hydrogels, microspheres, and nanofibers, has been shown to enhance nerve repair. The combination of multiple GFs in nerve conduits further improves axonal regeneration by mimicking the natural regenerative environment [120,132,154].

GFs exert their effects by binding to specific receptors and activating intracellular signaling pathways that regulate neuronal survival and axonal growth. Key pathways involved in peripheral nerve regeneration are summarized in Table 3.

The discovery of NGF dates to the 1950s and was recognized with the Nobel Prize in 1986 [155,156].

Nerve growth factors play a crucial role in the regeneration and repair of peripheral nerve injuries by modulating neurotrophin signalling pathways. NGFs primarily interact with two receptor types—the tropomyosin receptor kinase A (TrkA) and the p75 neurotrophin receptor (p75NTR)—which together regulate neuronal survival, differentiation, and axonal outgrowth. The formation of a high-affinity TrkA-p75 complex significantly enhances NGF binding, amplifying pro-survival signalling and neural plasticity. In cases of peripheral nerve damage, reduced NGF availability or impaired receptor function can lead to inadequate neuronal support, contributing to axonal degeneration and functional deficits. Moreover, the intricate balance between TrkA-mediated trophic signalling and p75NTR-induced apoptosis determines the regenerative capacity of injured nerves. Recent research suggests that modulating these receptor interactions and enhancing NGF bioavailability could be potential therapeutic strategies for improving nerve repair. Understanding the molecular mechanisms governing NGF signalling, including allosteric modulation and receptor crosstalk, is essential for developing targeted interventions to enhance peripheral nerve regeneration [132,157].

Glial-cell-line-derived neurotrophic factor (GDNF) is a neurotrophic factor that supports motor neuron survival, regeneration, myelination, and neuromuscular junction remodelling. It is upregulated by Schwann cells after nerve injury and secreted by skeletal muscle to prevent atrophy. Despite its therapeutic potential, GDNF has a short half-life and poor tissue penetration, complicating its clinical application. Persistently elevated levels can impair regeneration by causing axonal coiling and hypertrophy. While precisely timed delivery enhances motor neuron survival and axonal regeneration, gene therapy provides a more sustained local GDNF source through viral-vector-transduced cells. However, excessive expression still leads to axonal coiling, highlighting the importance of controlled timing, dosage, and localization. Biocompatible nerve conduits incorporating GDNF, such as silk-fibroin-based conduits with NGF and GDNF, show promise in improving neuroprotection and axon regeneration [158,159,160].

Fibroblast Growth Factor (FGF-2) enhances Schwann cell proliferation, migration, and differentiation. It interacts with FGFR1 to activate MAPK/ERK and PI3K/Akt pathways, facilitating nerve repair and remyelination [132].

Platelet-rich plasma (PRP) therapy is being explored for peripheral nerve injury due to its growth-factor-rich composition, which promotes cell proliferation, differentiation, and tissue regeneration. Animal studies show PRP accelerates nerve regeneration, while in vitro research confirms its role in enhancing Schwann cell activity. Clinical trials for conditions like carpal tunnel syndrome and nerve grafting yield mixed results, likely due to variations in PRP preparation. With its autologous origin, cost-effectiveness, and regenerative potential, PRP shows promise for nerve repair, but further standardization and research are needed for clinical application [161,162,163].

GF-based therapies represent a significant advancement in peripheral nerve injuries treatment, particularly when combined with biomaterial-based delivery systems. The controlled and sustained GF release through nerve conduits enhances axonal regeneration, reduces scar formation, and improves functional recovery. Future research should focus on optimizing biomaterial properties, investigating the synergistic effects of multiple GFs, and advancing clinical translation to improve outcomes for patients with severe nerve injuries [132,164].

Another encouraging delivery system is gene therapy that distributes growth factors through viral vectors or nanoparticles [160].

Recent studies have demonstrated that transplanting engineered Schwann cells with doxycycline-inducible GDNF expression promotes axonal growth, whereas persistent GDNF expression hinders regeneration. Although this system allows precise gene regulation, its long-term effectiveness in rodents and non-human primates is compromised by an immune response against the rtTA transactivator, resulting in the elimination of transduced cells. To address this limitation, researchers have developed an immune-evasive doxycycline-inducible GDNF gene switch (dox-i-GDNF) to ensure sustained and controlled GDNF expression [160,165,166,167].

Clustered Regularly Interspaced Short Palindromic Repeats (CRISPR) is a groundbreaking gene-editing technology that has been adapted for various purposes, including gene activation (CRISPRa). CRISPR also enables multiplexing, meaning multiple genes can be targeted simultaneously by designing different guide RNAs for each target. Gene therapy for nerve regeneration often involves the delivery of complementary DNA (cDNA) encoding specific neurotrophic factors, which, when overexpressed, can significantly enhance nerve repair and regeneration. Notably, Hsu et al. used the CRISPRa system for the synergic activation of multiple neurotrophic factors, such as BDNF, GDNF, and NGF [168,169].

Sterile alpha and toll/interleukin-1 receptor motif containing 1 (SARM-1) is a protein involved in regulating Wallerian degeneration, which makes it a promising target for therapeutic interventions aimed at blocking this degenerative process. Small-molecule inhibitors targeting SARM1 are currently being developed with the potential to slow axonal degradation following peripheral nerve injury, such as the isoquinoline inhibitor DSRM, as described in a study by Hughes et al. [170,171,172].

### 4.4. The Role of Stem Cells in Peripheral Nerve Regeneration

Another topic of interest regarding nerve regeneration and axonal regrowth is the inclusion of stem cells in nerve conduits. Mesenchymal stem cells are pluripotent cells that can transform into different types of cells. There are multiple sources of mesenchymal stem cells, such as bone marrow, umbilical cord, or adipose tissue. The application of mesenchymal stem cells to nerve conduits is beneficial due to their ability to differentiate into Schwann-like cells and produce neurotrophic factors, which promote improved regeneration. Another advantage of mesenchymal stem cells is their capacity to migrate to the lesion site and produce bioactive agents, reducing the inflammatory response. Incorporating stem cells into nerve conduits—either by directly adding them inside the conduit in various solutions or gels, or by embedding them within the nerve conduit as cell-loaded scaffolds—has demonstrated enhanced nerve regeneration. Numerous studies have shown significant improvements in nerve regeneration when mesenchymal stem cells are added to nerve conduits, compared to conduits without stem cells [3,147,148,149,150,151,152].

An experimental study reported by di Summa et al. aimed to enhance the fibrin nerve conduits by incorporating regenerative cells. The study evaluated different cell populations, including primary Schwann cells and stem cells differentiated into a Schwann cell-like phenotype, to determine their effectiveness in supporting nerve repair. The results demonstrated a significant improvement in axonal regeneration in the fibrin conduit seeded with Schwann cells compared to the empty fibrin conduit. Additionally, differentiated adipose-derived stem cells promoted regeneration distances comparable to those achieved with differentiated bone marrow mesenchymal stem cells. These findings suggest that adipose-derived stem cells could serve as a viable alternative without the donor site morbidity associated with Schwann cell harvesting [173].

For future clinical applications, adipose-derived stem cells offer a significant advantage due to the abundant subcutaneous fat deposits in humans. These stem cells can be harvested in large quantities using a minimally invasive liposuction procedure under local anaesthesia, which eliminates the discomfort and tissue morbidity associated with bone marrow aspiration. These characteristics make adipose-derived stem cells a promising alternative to mesenchymal stem cells for tissue engineering applications and peripheral nerve repair [173,174].

A study by Chen et al. presents a modified protocol for generating three-dimensional (3D) Schwann-like cell (SLC) spheroids from adipose-derived stem cells using a recombinant peptide scaffold in order to enhance differentiation efficiency and neurotrophic factor secretion. Morphological evaluation, gene expression, and functional assays demonstrated that 3D SLCs exhibit superior Schwann cell marker expression, neurotrophic factor secretion, and neurite outgrowth promotion compared to conventional two-dimensional cultures. In a sciatic nerve injury mouse model, the transplantation of 3D SLCs significantly improved axonal regeneration, motor function recovery, and muscle preservation, indicating their enhanced therapeutic potential. These findings suggest that 3D SLC spheroids offer a clinically translatable, scalable, and immunologically favorable approach for peripheral nerve repair, overcoming limitations associated with primary Schwann cell isolation and two-dimensional differentiation systems [175].

Masgutov et al. reported a study exploring the use of fibrin glue as a delivery method of adipose-derived mesenchymal stem cells in peripheral nerve repair, providing both cell fixation and extracellular matrix support. Using a sciatic nerve injury model, adipose-derived MSCs embedded in fibrin glue successfully migrated into the nerve, exerting neuroprotective effects on sensory neurons, promoting axonal growth and myelination, enhancing angiogenesis and motor function recovery. These findings suggest that fibrin-glue-based MSC therapy is a promising and effective approach for peripheral nerve regeneration [176].

Another investigated strategy involves the combination of nerve allografts with stem cell therapy to enhance peripheral nerve regeneration. The vascularization of processed and decellularized allograft nerves improves with the incorporation of both undifferentiated and differentiated mesenchymal stem cells. Revascularization in these grafts, regardless of mesenchymal stem cell presence, primarily occurs through centripetal revascularization, whereas autograft nerves undergo vascularization predominantly via inosculation [177].

In an experimental model on Lewis rats, Mathot et al. showed that both undifferentiated and differentiated mesenchymal stem cells significantly enhanced functional recovery in decellularized allografts at 12 weeks, yielding results comparable to autografts across most assessments. By 16 weeks, outcomes stabilized as anticipated. While the differences between the two cell types were not statistically significant, undifferentiated mesenchymal stem cells demonstrated a greater capacity to improve functional outcomes and offered practical advantages for clinical application by reducing preparation time and associated costs [178].

Adipose-derived mesenchymal stem cells demonstrate promising potential when combined with decellularized allografts in multiple experimental studies on peripheral nerve regeneration [177,179].

Saffari et al. investigated the impact of surgical angiogenesis and stem cell therapy on the microvascular architecture of nerve allografts in a rat sciatic nerve defect model. Using microcomputed tomography, the vascular volume and vessel distributions were analyzed across five experimental groups: autografts, allografts, allografts with surgical angiogenesis, meaning wrapping the allograft in superficial inferior epigastric artery fascia flap, and allografts combined with either undifferentiated or Schwann cell-like mesenchymal stem cells. The results demonstrated that allografts treated with both surgical angiogenesis and undifferentiated MSCs exhibited the greatest vascular volume and vessel diameter, significantly surpassing all other groups. This combination not only enhanced vascularization but also facilitated vessel penetration into the mid-longitudinal segment of the nerve graft, suggesting a synergistic effect of surgical angiogenesis and undifferentiated MSCs in promoting revascularization and potential nerve regeneration [180].

Koplay et al. explored the effects of adipose-derived mesenchymal stem cells and adipose-derived-mesenchymal-stem-cell-originating exosomes on nerve allograft regeneration in a rat experimental model and demonstrated favorable outcomes for nerve regeneration. In cellularized allografts, the exosome-treated group showed significantly improved axon–myelin regeneration, endoneural connective tissue organization, and reduced inflammation. In decellularized allografts, MSC and exosome treatments resulted in better electromyographic outcomes, including reduced latency and enhanced action potential at later stages. A histo-morphological analysis also revealed improved vascularization in these experimental groups [181].

Extracellular-vesicle-based therapy, particularly exosome therapy, presents a promising approach for tissue regeneration. Exosomes facilitate intercellular communication and homeostasis by transferring proteins and nucleic acids through endocytosis, influencing key cellular pathways. Their regenerative benefits have also been demonstrated in neural tissues, with a notable role in neuroprotection and axonal regeneration. Exosomes derived from bone marrow mesenchymal stem cells, umbilical cord MSCs, adipose-derived stem cells, and gingiva-derived MSCs have shown potential in enhancing nerve repair and functional recovery. This approach presents a clinically translatable strategy for advancing peripheral nerve repair and developing new regenerative therapies [182,183,184,185,186,187].

MSC-derived exosomes serve as key paracrine mediators in peripheral nerve repair by enhancing angiogenesis, axonal regeneration, neuroinflammation modulation, and neuropathic pain relief. These extracellular vesicles are enriched with bioactive molecules, including VEGF, PDGF-D, and miRNAs (e.g., miR-1260a, miR-21-5p, and miR-29b-3p), which promote endothelial cell proliferation and vascular regeneration via the PI3K/AKT and AKT/eNOS pathways. Exosomes from bone marrow MSCs (BMSCs), adipose-derived MSCs (ADMSCs), and umbilical cord MSCs (UCMSCs) stimulate Schwann cell dedifferentiation and axonal regrowth through pathways like c-JUN and MEK/ERK. Engineered exosomes enriched with miR-26a, miR-133b, and the miR-17-92 cluster further enhance axonal outgrowth and neuronal survival. Additionally, the exosome-mediated modulation of macrophage polarization (M1 to M2) through the TLR4/NF-κB/STAT3/AKT axis reduces neuroinflammation, while let-7b and miR-181c-5p downregulate TNF-α and IL-1β, contributing to neuropathic pain relief by increasing IL-10 and BDNF expression [185,187].

To optimize exosome-based therapy, biomaterial integration enhances their localized and sustained release at injury sites. Nerve conduits, such as chitosan and electrospun poly(lactic-co-glycolic acid) (PLGA) scaffolds, combined with MSC exosomes, improve axonal regeneration and vascularization while reducing neuroma formation. Studies using conductive hydrogels (ECHs) loaded with BMSC-derived exosomes demonstrate improved Schwann cell adhesion and nerve conduction via NF-κB and MEK/ERK activation. Thermosensitive hydrogels, such as hydroxyethyl chitosan/β-glycerophosphate matrices, enable controlled exosome delivery, accelerating peripheral nerve repair. By leveraging exosome-loaded biomaterials, nerve regeneration strategies achieve superior outcomes, facilitating functional recovery through molecular signalling modulation and sustained neurotrophic support [182,183,184,185,186].

Olfactory ensheathing cells (OECs) are specialized glial cells originating from the neural crest that play a crucial role in facilitating the growth and regeneration of primary olfactory neurons. The primary olfactory system exhibits continuous neurogenesis throughout adulthood, a phenomenon largely attributed to the neuroprotective and pro-regenerative microenvironment maintained by OECs. This intrinsic capacity for neural repair has been leveraged in cellular transplantation strategies, particularly in experimental models of spinal cord injury, where OECs have demonstrated potential in promoting axonal regeneration and functional recovery [188,189].

The role of OECs was also explored for improving peripheral nerve repair. The regenerative potential of olfactory ensheathing cells is mediated through multiple mechanisms, including the secretion of neurotrophic factors, neuroprotection, the modulation of the inflammatory response, and the promotion of axonal regeneration. OECs facilitate the guidance of newly formed axons across the injured microenvironment, bridging the gap between both ends of an injured nerve. Furthermore, OEC transplantation demonstrates the capacity for targeted migration and the penetration of glial scars, thereby enhancing the repair and functional recovery of damaged neural pathways [189,190,191,192].

OEC cellular transplantation in various experimental rat models, including sectioned recurrent laryngeal nerve and sciatic nerve injuries, showed promising results in improving nerve regeneration [189,193].

Researchers have enhanced OEC function in peripheral nerve injury by combining them with other active cells or biomaterials, achieving promising results [189].

Embedding the OECs in a poly (ε-caprolactone) (PCL) conduit used for sciatic nerve repair in a rat model showed a regenerative result comparable to the use of an autologous nerve graft, offering a valuable reconstructive option [194].

Co-culturing OECs with human-umbilical-cord-mesenchymal-stem-cell-derived exosomes enhanced OEC survival, migration, and brain-derived neurotrophic factor expression under hypoxia. This synergy promoted nerve regeneration and functional recovery, suggesting a potential therapeutic approach for severe nerve injuries in hypoxic conditions [195].

### 4.5. Pharmacological and Bioactive Compound Interventions for Peripheral Nerve Repair

Although we do not have any medically approved pharmacological treatment in use for peripheral nerve regeneration, research in the field of molecular biology has given us some directions towards influencing different metabolic processes that are involved in nerve regeneration following injury. Various pharmacological agents have been tested in experimental trials aiming to promote axonal growth, reduce inflammation, and scarring in order to achieve a better functional outcome. Several drugs, including vitamins (vitamin B), hormones (melatonin and erythropoietin), immunosuppressants (tacrolimus), and antioxidants (curcumin and lipoic acid), have demonstrated beneficial effects on nerve healing. Either by topical application or by systemic application, these pharmacological agents offer a large variety of potential adjuvants that can lead to a better functional recovery after peripheral nerve injuries. Their mechanisms of action, which range from promoting cell growth and relocation of growth factors to reducing inflammation, scarring, and protecting against oxidative damage, hold significant potential towards achieving better results in peripheral nerve regeneration [196,197,198,199,200,201].

#### 4.5.1. Tacrolimus

Tacrolimus, or FK506, is an immunosuppressive macrolide identified in 1984, and it was extracted from Streptomyces tsukubaensis. Initially developed for preventing organ rejection in transplants, tacrolimus has also been recognized for its beneficial effects on nerve regeneration. The compound’s potential on nerve regeneration was first identified in 1994 when it was found to stimulate sensory nerve growth even at very low concentrations in vitro. Additional studies in animal models have confirmed its ability to stimulate axon regrowth, increase the size and number of regenerating axons, and enhance myelination, all of which contribute to improved results in motor function [62,94,202,203,204].

Tacrolimus exerts its effects on nerve regeneration through its interaction with FKBP52, a protein essential for leading regenerating axons. After a nerve lesion, this protein structure relocates to the growth cones of injured neurons, helping in enhancing the regeneration process. The drug’s neuroregenerative effect has been demonstrated in both systemic and local applications, with studies showing enhanced nerve growth and the faster regeneration of peripheral nerves. Tacrolimus also helps in decreasing the development of scar tissue at injury sites, possibly by triggering apoptosis of the fibroblasts, preventing the accumulation of excessive fibrotic tissue. This action further supports the recovery of nerve function following injury [205,206,207,208,209].

Although the exact mechanisms behind tacrolimus’ effects on nerve regeneration remain unclear, its ability to stimulate nerve growth, reduce scarring, and promote quicker recovery makes it a promising therapeutic option for nerve injuries. However, variations in experimental outcomes highlight the need for more research to fully understand how tacrolimus aids in nerve repair [201,210].

#### 4.5.2. Calcium Channel Blockers

The latest research has emphasized the critical role of calcium in scar development. Calcium channel blockers can reduce fibroblast activity and collagen production, which helps reduce fibrotic tissue buildup. The accumulation of fibrotic tissue at the level of injury after nerve damage leads to neuroma formation and prevents nerve recovery. Studies have shown large concentration of calcium involved in this fibrotic tissue. Following a nerve lesion, calcium rapidly enters the damaged nerve due to the disruption of the myelin layer, leading to a dangerous increase in intracellular calcium levels. This increase in calcium levels triggers various secondary metabolic processes that lead to apoptosis of the Schwann cells and contribute to nerve degeneration [211,212,213,214,215,216,217].

The excessive activation of calcium-dependent processes results in the activation of enzymes such as protein kinases and proteases, leading to the production of harmful substances. These changes further activate genetic pathways that induce cellular apoptosis. Studies have shown a significant association between calcium levels and the severity of nerve injury as well as the success of recovery. Elevated calcium levels have been proven to induce the apoptosis of Schwann cells, limiting their development. This highlights the importance of maintaining normal calcium concentrations for cellular health and proper nerve regeneration [212,218,219,220,221].

#### 4.5.3. Statins

Statins, primarily known for lowering cholesterol, have shown promising results in neurological recovery due to a wide range of effects, including neuroprotection, inflammation reduction, and combating oxidative stress. Drugs like simvastatin and rosuvastatin help facilitate nerve regeneration and recovery after injuries by stimulating stem cell relocation and increasing neurotrophic growth factors to the injury site. These properties have led to increased interest in using statins for various neurological conditions, including Parkinson’s disease or Alzheimer’s disease, as well as their use in peripheral nerve injuries. There are multiple studies, conducted mainly on rats, that show the positive effects of statins on nerve regeneration, both from oral and topical applications [222,223,224,225,226,227,228].

#### 4.5.4. Lipoic Acid

Lipoic acid is a compound produced within our bodies that can be supplemented through diet, with potent antioxidant properties. Lipoic acid prevents oxidative damage by reducing lipid degradation and free radical production, thereby helping to prevent cell apoptosis. Several studies, conducted on animal models, have shown that lipoic acid plays a beneficial role in nerve regeneration following nerve lesions, improving axonal regrowth, myelination, and nerve conduction. Some research showed a better functional outcome from groups treated with alpha-lipoic acid than vitamin B12. This highlights the potential of lipoic acid as a therapeutic agent for improving peripheral nerve regeneration after injury [200,229,230,231,232].

#### 4.5.5. Vitamin B

Multiple physiological functions, including DNA and RNA formation, various biochemical processes, and immune protection, are critically influenced by vitamins from the B complex, especially vitamins B1 (thiamine), B6 (pyridoxine), and B12 (cobalamin). Cobalamin is a vitamin that is hydrosoluble and can be found in milk products, eggs, or meat. It acts as a coenzyme and helps the production of methionine from homocysteine, which is crucial for protein and nucleic acid formation. This process supports several vital functions involved in the regeneration of the nervous system by stimulating axonal regrowth and enhancing the synthesis of myelin. Additionally, vitamin B12 is involved in decreasing the degree of endoplasmic reticulum stress. When a nerve lesion occurs, the stress levels in the endoplasmic reticulum increase, and, combined with inflammation, this results in neuronal damage. Vitamin B12 plays a role in increasing the brain-derived neurotrophic factor and nerve growth factor and boosts the metabolism of proteins, thus stimulating axonal regeneration. The administration of a vitamin B cocktail (B1, B6, and B12) has been associated with a decrease in nerve degeneration. A large supply of vitamin B12 can increase axonal regrowth and enhance the functional results following peripheral nerve injuries [201,233,234,235,236,237,238,239,240,241].

#### 4.5.6. Erythropoietin

Erythropoietin (EPO) is a well-known pro-angiogenic factor that also possesses anti-inflammatory, anti-apoptotic, anti-oxidative, phagocytic, neurotrophic, and neuroprotective properties. Research has demonstrated its ability to enhance peripheral nerve regeneration and functional recovery, because it preserves and promotes Schwann cell myelination, improves myelin thickness and axon diameter, and increases the total number of regenerated nerve fibers. Additionally, EPO contributes to early angiogenic responses following nerve injury, supporting vascularization and tissue repair. To optimize the therapeutic application of EPO, Manto et al. [242] developed an EPO-loaded thermogel (EPO-PLGA-PEG), composed of amphiphilic polyethylene glycol/poly lactic acid-co-glycolic acid (PEG/PLGA) polymers. This gel remains liquid at room temperature but solidifies in situ at body temperature, allowing for sustained, localized EPO release over approximately three weeks. Importantly, this controlled-release system demonstrated no adverse hematologic effects, while promoting both neurogenesis and angiogenesis at the injury site. Sundem et al. showed that the local administration of EPO in a fibrin-glue carrier provided comparable neuroregenerative benefits with the systemic administration of EPO, without the risks associated with systemic administration [243,244].

#### 4.5.7. Melatonin

Melatonin, a hormone produced by the pineal gland, is involved in various physiological processes, such as the sleep–wake cycle, cognitive functions, reproductive functions, and aging. It is also known for its neuroprotective properties, particularly in the context of nerve regeneration. Melatonin has been shown to support the integrity of myelin sheaths, enhance nerve regeneration, and promote functional recovery following peripheral nerve injury. Its positive impact is linked to its potent antioxidant effect, which helps combat oxidative stress by neutralizing free radicals. Additionally, melatonin has been found to increase the recruitment of Schwann cells at the site of the nerve injury and accelerate axonal regrowth, all contributing to improved nerve conduction and recovery. Studies in animal models, particularly rats, have demonstrated that melatonin administration enhances axon growth following traumatic nerve injuries, while decreasing the risk of developing a neuroma and the proliferation of fibrotic tissue [200,201,245,246,247,248,249,250].

#### 4.5.8. Hyaluronic Acid

Hyaluronic acid (HA) is a widely spread polysaccharide that plays an essential role in forming the extracellular matrix. Due to its biodegradable properties and biocompatibility, generated by the lack of specificity between species, producing a minimal immune response, HA is considered a promising material for aiding regeneration following peripheral nerve injuries. Research has shown that HA can significantly limit scar tissue and nerve adhesions after neurolysis or other forms of nerve injuries, thereby supporting nerve regeneration [251,252,253,254].

The effect of HA on axonal growth is influenced by the dose, with higher concentrations leading to improved axonal regrowth. Additionally, combining HA with growth factors like nerve growth factor (NGF) and vascular endothelial growth factor (VEGF) may further enhance nerve regeneration through a joint influence on the neuronal microenvironment. Overall, HA-based biomaterials hold significant potential for promoting nerve regeneration by providing an optimal setting for neural growth and restructuring the fibrin matrix [201,246,255,256,257].

#### 4.5.9. Curcumin

Curcumin, the primary compound found in turmeric, has been widely used for its anti-inflammatory and analgesic properties. Its ability to reduce inflammation and oxidative processes is believed to occur by downregulating the mitogen-activated protein kinases. Beyond its pain-relieving and anti-inflammatory properties, curcumin also demonstrates neuroprotective effects that could aid in nerve regeneration. Studies have shown it can reduce cell apoptosis, enhance nerve conduction and sensitivity, stimulate axonal growth, and promote myelination in rat models. Additionally, curcumin helps by decreasing free radicals and by improving the size of nerve fibers, making it a promising agent for supporting nerve regeneration and recovery after injury [200,233,258,259,260,261,262,263].

### 4.6. The Role of Electrical Stimulation in Nerve Regeneration

Electrical stimulation (ES) is an adjunctive instrument for enhancing peripheral nerve regeneration. Following peripheral nerve injury, ES has been shown to facilitate the early phases of nerve repair, including promoting neuronal survival and the formation of axonal sprouts [264].

The primary mechanisms involved in peripheral nerve regeneration are associated with an enhancement in neurotrophic factor expression, both at the neuronal level as well as at the Schwann cell level, leading to changes in the microenvironment at the injury site. Electrical impulses generate an influx of growth factors such as brain-derived neurotrophic factor (BDNF) and nerve growth factor (NGF), which are critical for axonal regrowth and rebuilding synapses [265].

**Figure 4:**
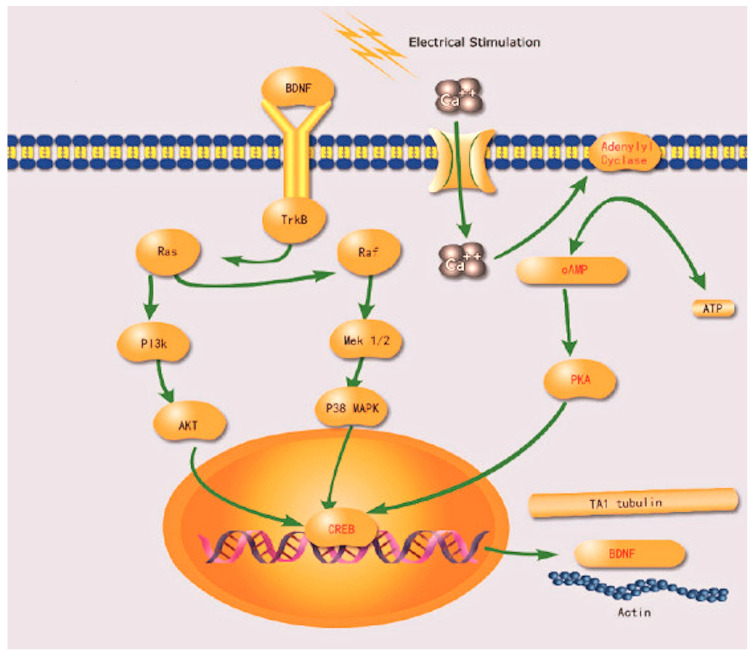
Effect of electrical stimulation on the expression of regeneration-associated genes within the neuronal cell body of peripheral nerves (adapted from Chu XL et al. [266], under CC BY-NC 4.0 licence).

In addition to upregulating neurotrophic factors, ES affects intracellular signalling pathways that are vital for nerve regeneration. Notably, ES has been found to activate the phosphatidylinositol 3-kinase/Akt (PI3K/Akt) and mitogen-activated protein kinase (MAPK) pathways. The activation of the PI3K/Akt pathway supports cell survival and growth, while the MAPK pathway is involved in cellular responses to growth signals. These pathways collectively contribute to the regeneration process by enhancing axonal elongation, as shown in Figure 4 [266].

Furthermore, electrical stimulation has been shown to upregulate the expression of genes involved in cytoskeletal rearrangement, thereby facilitating axonal extension and alignment through the Schwann cell bands of Büngner. Different studies have shown that ES generates a boost in various protein production, such as S100B, GFAP, NGFR, MBP, and MPZ, leading to an increase in myelin formation and promoting axonal growth [267,268].

Clinical and preclinical models have revealed that the optimal frequency, intensity, and duration of stimulation are crucial factors influencing regenerative outcomes. Low-frequency stimulation (20 Hz) has been associated with enhanced motor recovery. For example, brief low-frequency ES accelerates motor and sensory axon regrowth across injury sites, even after the delayed surgical repair of injured peripheral nerves. High-frequency ES has been shown to better augment nerve regeneration compared to low-frequency ES; however, using high frequencies can induce neuropathic pain, especially if used right after the nerve repair. This setback can be improved by delaying the initiation of the electrical stimulation. Furthermore, continuous high-frequency stimulation may cause detrimental effects, including axonal fatigue [269,270].

Recent advancements in bioelectronic interfaces and implantable devices have further enabled the precise control of ES parameters, allowing tailored interventions for individual patients. For instance, the development of biodegradable and flexible neural implants allows for wireless transdermal optoelectronic stimulation for neuromodulation, providing a means to deliver controlled ES in a minimally invasive manner [271].

Overall, electrical stimulation represents a viable adjunctive therapy in nerve repair strategies, enhancing natural regenerative mechanisms and improving clinical outcomes.

ES also provides valuable information about the dynamic changes occurring during the injury and recovery process. Recent studies have demonstrated that applying pulsed ES to the injured muscle, combined with monitoring muscle fiber fluctuations—referred to as the muscle velocity recovery cycle (MVRC)—yields critical in vivo information about the depolarized resting potential following peripheral nerve injury. This method enhances our understanding of the underlying mechanisms of muscle weakness caused by nerve injuries, which is associated with a drop in muscle excitability [264,272].

## 5. Clinical Algorithm for Selecting Reconstructive Methods for Peripheral Nerve Transections

Primary repair is the preferred approach for peripheral nerve injuries when performed within the first few days. In contrast, secondary repair is undertaken a week or more after the injury. Partial nerve injuries, which account for approximately 15% of cases and often result from stretching or contusions, are typically managed with secondary repair [56,273].

In cases of complete nerve transection, the choice of repair method depends on intraoperative findings. If the epineurium is neatly sectioned, a tension-free primary repair is usually performed. However, if the nerve ends are irregular or severely contused, the interposition of a nerve graft or nerve conduit may be required to restore continuity and optimize functional recovery [56].

The selection of a technique for bridging a gap in peripheral nerves is primarily influenced by the length of the gap, potential donor site morbidity from graft harvesting, surgical duration, and the expertise of the operating surgeon. Table 4 analyzes nerve repair techniques that address nerve defects, highlighting their key advantages and limitations [8,56,273].

The repair of peripheral nerves depends on the affected anatomical area and the complexity of the nerve defect.

Nerve surgery in the maxillofacial region primarily involves trigeminal and facial nerves. Trigeminal nerve injuries commonly result from trauma, local anesthesia, tumor excision, implant placement, or, most frequently, third molar extraction, particularly affecting the inferior alveolar and lingual nerves. While primary repair at the time of injury is ideal, many injuries go unnoticed until after surgery. Early secondary repair, around three months post-injury, is widely accepted, though satisfactory outcomes can still be achieved later. Direct end-to-end anastomosis yields the best results, but, if a gap exists, a tension-free nerve graft is required to facilitate axonal regeneration. Common grafting materials include autologous sources (like sural and greater auricular nerves), vein grafts acting as conduits, and biodegradable allografts like the polyglycolic-acid-based neurotube [274,275,276].

Facial nerve repair is critical for restoring neuromuscular function following trauma, iatrogenic injury, tumor resection, or infection-induced paralysis. The choice of repair technique is determined by the severity and acute or chronic type of injury. Direct epineural or perineural neurorrhaphy is the preferred method for clean transections with minimal tension, ensuring optimal axonal regeneration. In cases where a nerve gap is present, autologous nerve grafts (e.g., sural or great auricular nerve), decellularized allografts, or synthetic nerve conduits are employed to maintain a tension-free repair and provide a scaffold for axonal regrowth. Fibrin glue can be used for nerve repair, replacing the nerve suture in indicated cases [58,277,278].

For proximal or chronic nerve injuries where primary repair is unfeasible, nerve transfers utilizing adjacent motor nerves, such as the masseteric or hypoglossal nerve, facilitate functional recovery by redirecting neural input to denervated facial muscles. In extensive nerve defects or longstanding facial paralysis, cross-facial nerve grafting followed by free functional muscle transfer is employed to restore dynamic reanimation. Additionally, regenerative strategies, including stem cell therapy, neurotrophic factor augmentation, and bioengineered nerve scaffolds, are under investigation to enhance axonal regeneration and improve clinical outcomes in facial nerve reconstruction [58,277].

A particular case is the functional recovery of patients who have undergone face transplantation. Motor recovery and the management of facial reconstruction are essential for restoring the quality of life in these patients [279].

The reconstruction of upper limb peripheral nerves employs a multimodal approach integrating direct nerve repair, nerve grafting, nerve transfers, and bioengineered scaffolds, with the primary objective of optimizing neural regeneration and functional recovery. Direct end-to-end epineural coaptation remains the preferred technique when feasible, ensuring tension-free repair to facilitate optimal axonal regrowth. However, in cases of extensive nerve gaps, alternative strategies such as autologous nerve grafting, processed nerve allografts, and nerve conduits are used to bridge defects and restore sensory and motor function. Autologous nerve grafts, typically harvested from the sural nerve, medial antebrachial cutaneous nerve, or posterior interosseous nerve, remain the gold standard due to their preserved Schwann cells and extracellular matrix, which provide an ideal scaffold for axonal regrowth. However, they are associated with donor site morbidity and limited availability. To address these limitations, processed nerve allografts, which are derived from decellularized human cadaveric nerves, have emerged as a viable alternative. These allografts retain critical structural components such as basal lamina and laminin, supporting axonal regeneration in nerve gaps of up to 3 cm with functional outcomes comparable to autologous grafts. Nerve conduits, whether synthetic (polyglycolic acid and polyvinyl alcohol) or biologic (collage and porcine submucosa), provide a controlled environment for nerve regeneration and are primarily recommended for shorter digital nerve gaps (<10 mm) [62,273,280].

For complex injuries where nerve repair or grafting is insufficient, nerve transfers serve as an effective reconstructive strategy to restore motor and sensory function while minimizing donor site morbidity. Advances in axonal mapping and peripheral nerve imaging have enabled the refinement of distal nerve transfers, optimizing donor–recipient pairings for targeted reinnervation. Motor nerve transfers are particularly valuable in cases of high radial, ulnar, and median nerve injuries, facilitating faster recovery by bypassing proximal nerve lesions. Sensory nerve transfers are also gaining traction for restoring protective sensation, especially in the hand, where early reinnervation is critical for functional recovery. Additionally, end-to-side coaptation techniques allow for axonal sprouting from intact donor nerves, providing a supplementary mechanism for nerve regeneration while preserving donor function [62,273,281].

Emerging technologies, such as 3D-printed nerve conduits with bioactive growth factors and advanced neuroimaging modalities (MR neurography and diffusion tensor imaging), hold promise for enhancing nerve regeneration and optimizing patient outcomes. As research progresses, multimodal strategies integrating nerve grafting, transfers, and engineered biomaterials are likely to further improve functional recovery in upper limb peripheral nerve injuries [273,282].

For amputee patients, the vascularized composite allotransplantation of the upper limb represents an advanced therapeutic option, offering the potential to restore the functionality and sensorimotor integration of the lost limb. This complex intervention requires meticulous neurovascular reconstruction, including peripheral nerve repair and regeneration to enable the reinnervation of muscular and sensory structures. The success of the transplantation depends on an integrated approach combining microsurgical techniques, immunosuppressive therapy, and intensive rehabilitation programs to optimize the functional reintegration of the transplanted limb and improve the patient’s quality of life [283,284,285,286].

An alternative to vascularized composite allotransplantation is represented by targeted muscle reinnervation (TMR) technologies and advanced prosthetic systems. TMR enhances neural signal transmission by redirecting residual peripheral nerves to alternative muscle targets, allowing for more intuitive and precise prosthetic control. When combined with high-performance prostheses featuring myoelectric sensors, haptic feedback, and adaptive control algorithms, this approach offers significant functional restoration. Compared to transplantation, TMR with state-of-the-art prostheses eliminates the risks associated with immunosuppression while providing a viable solution for upper limb amputees. Continuous advancements in neuroprosthetics and bionic integration are further improving dexterity, sensory perception, and user adaptability, making this a compelling alternative for functional rehabilitation [286,287,288].

Peripheral nerve injuries in the lower limb, particularly those affecting the peroneal nerve, pose significant challenges due to their high incidence and poor prognosis. These injuries can result from trauma, fractures, iatrogenic causes, or compression, with the peroneal division of the sciatic nerve being the most frequently affected. Diagnosis relies on clinical examination, electrodiagnostic studies, and imaging modalities such as MRI and ultrasonography to assess the severity and location of the lesion. Treatment options include neurolysis, direct nerve repair, autologous nerve grafting, and, in some cases, nerve transfers. However, nerve transfers in the lower limb are less commonly performed and yield less favorable outcomes compared to the upper limb. The peroneal nerve’s vulnerability stems from its anatomical tethering, limited mobility, lower connective tissue content, and longer regeneration distance. Despite surgical interventions, functional recovery remains suboptimal, particularly for the peroneal division, highlighting the need for early intervention and advancements in nerve reconstruction techniques [289,290].

For foot and ankle regions, reconstructive approaches rely on direct nerve repair, autologous nerve grafts, nerve conduits, and processed nerve allografts to promote functional recovery [291,292,293].

**Figure 5:**
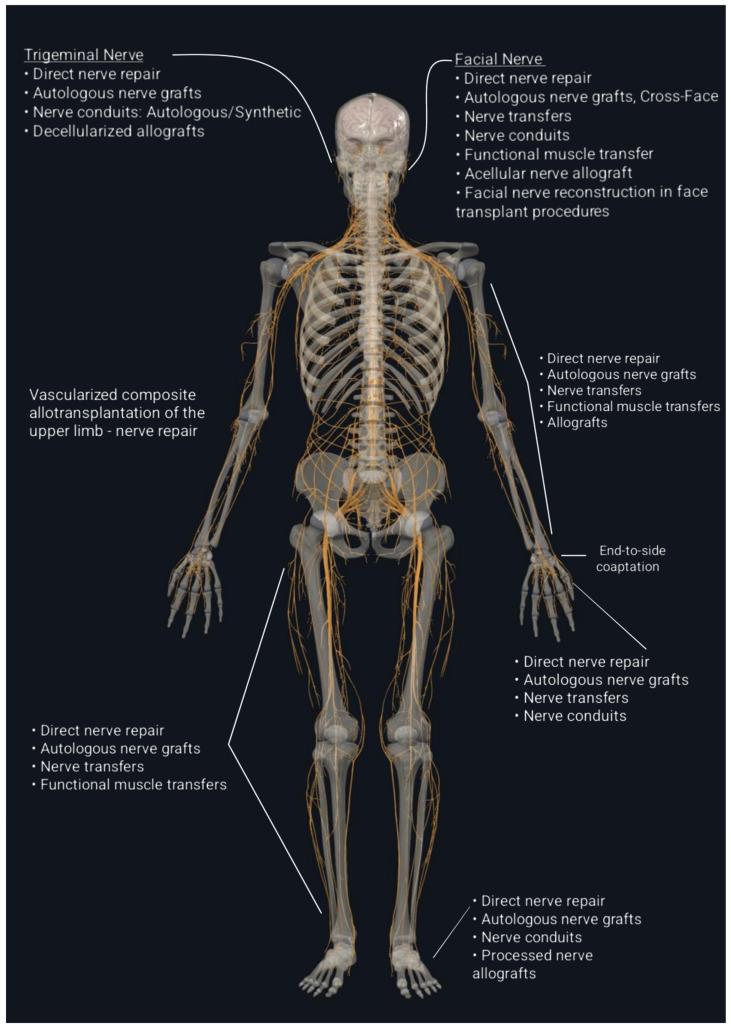
Therapeutic approaches for managing peripheral nerve injuries affecting the facial and limb regions.

Figure 5 depicts the aforementioned concepts, illustrating the therapeutic approaches that can be used to manage peripheral nerve injuries affecting the face and limbs. These injuries are clinically significant due to the substantial functional impairments they induce.

## 6. Examples of Nerve Repair Strategies in Clinical Application

Despite the extensive body of research on peripheral nerve regeneration, many therapeutic strategies have yet to be implemented in clinical practice. Direct nerve repair using epineural microsutures remains the gold standard surgical approach for severe axonotmesis and neurotmesis injuries [62].

When a gap exists between the nerve ends that creates excessive tension for direct epineural repair, reversed interposition autologous nerve grafts are necessary. Human autografts are preferred, as evidence demonstrates that autografting is superior to nerve conduits for longer gaps (greater than 3 cm), more proximal injuries, and critical nerves [62,294].

Human cadaveric nerve allografts have been utilized in select cases involving extensive nerve injuries where suitable autologous donor tissue is unavailable. In such instances, recipients typically undergo immunosuppressive therapy for up to two years, allowing time for the donor graft to become repopulated with host Schwann cells. According to Moore et al., nerve allotransplantation should be considered only for exceptional cases in which untreated peripheral nerve injuries would otherwise result in a severely impaired or nonfunctional limb [62,295].

Nerve repair coaptation is a crucial part of vascularized composite allotransplantation, as it enables peripheral nerve regeneration needed to restore function in transplanted tissues. Tacrolimus, a standard-of-care immunosuppressive agent commonly used in vascularized composite allotransplantation, has also been shown to promote nerve regeneration [210,296].

Alternative approaches to traditional direct suture repair or autologous nerve grafting have gained attention as potential substitutes. One of the primary challenges in nerve repair is directing regenerating sensory, motor, and autonomic axons toward the distal, degenerating nerve segment to enhance the likelihood of successful reinnervation at the proximal end. To address this, several options have been introduced, including allogeneic processed grafts, hollow nerve conduits, and coaptation devices, all of which show promise in the reconstruction of severed peripheral nerves [297,298]. Table 5 presents the key characteristics of a series of commercially available nerve conduits currently used in clinical practice.

Nerve allografts have been decellularized through processes involving chemical detergents, enzymatic degradation, and irradiation, resulting in grafts that do not require immunosuppression. One advantage of these clinically available grafts over hollow nerve conduits is the preservation of internal nerve architecture, including endoneurial tubes, basal lamina, and laminin which supports axonal regeneration [62,309]. A level III study published by Cho et al. evaluated the safety and efficacy of using processed nerve allografts (Avance Nerve Graft) for reconstructing peripheral nerve injuries in the upper extremity, as part of the multicenter RANGER Study registry. Analyzing 71 nerve repairs in 56 patients, with gap lengths ranging from 5 to 50 mm, the study found that 86% of procedures resulted in meaningful functional recovery (defined as ≥S3 for sensory or ≥M4 for motor function). The subgroup analysis showed strong outcomes across sensory, mixed, and motor nerves, with digital nerve repairs showing the highest success (89%). No complications related to the grafts were reported [310].

A multicenter registry study published by Safa et al. presents a large dataset on the use of processed nerve allografts, specifically the Avance^®^ Nerve Graft, for peripheral nerve repair. Drawing from 1630 nerve repairs across 31 centers, the study evaluated 385 patients with 624 nerve repairs and found that 82% of cases achieved meaningful recovery (≥S3/M3), including repairs of sensory, mixed, and motor nerves with nerve gaps up to 70 mm. The study demonstrated consistent efficacy across various subgroups (nerve type, patient age, smoking status, and time to repair), with particularly strong outcomes in upper extremity repairs (83% recovery), digital nerves (84%), and shorter gaps (<30 mm). Repairs involving complex injuries or longer gaps (>50 mm) showed slightly lower recovery rates, although still comparable to historical autograft data and superior to nerve conduits. The use of processed allografts was shown to be safe, with a low adverse event rate (3.7%) by subjects and no product-related complications [311].

Currently, nerve conduits are not yet generally considered reliable alternatives to autologous nerve grafts for motor nerve repair, gaps longer than 3 cm, or proximal nerve injuries [62,312].

The cost represents a critical determinant in modern healthcare decision-making. Comparative analyses have been conducted to evaluate the economic implications of different nerve repair strategies, including autologous nerve grafting and the use of synthetic or biological nerve conduits. Processed allografts, nerve conduits, and wraps are substantially more expensive per unit compared to traditional microsurgical sutures used in nerve repair. Their widespread use could pose a significant financial burden on healthcare systems. Additionally, there is a significant difference in affordability between centers around the world, which may limit their use in low-resource settings. It has been shown that socioeconomic factors influence outcomes following the repair or reconstruction of digital or major nerve trunk injuries in the upper limb, anatomic areas with important functional requirements [280,298,313].

The interest regarding stem cell therapy has grown due to its potential benefit on nerve regeneration and has been clinically tested in various neurodegenerative diseases, such as Alzheimer’s disease or multiple sclerosis, and for traumatic injuries to the spinal cord. However, there are a small number of clinical trials focusing on the clinical aspects of stem cells in peripheral nerve injuries [314,315].

A study from Levi et al. has shown promising results in a couple of cases where autologous stem cells were used to improve nerve regeneration following sural nerve grafts. Both patients showed excellent motor and sensory recovery after the treatment [316]. Another research from Braga-Silva et al. has compared the results of 44 patients with median and ulnar nerve gaps following injury. Half of them were treated using silicone tubes to bridge the nerve gap. The second half was managed with silicone tubes filled with autologous stem cells obtained from the iliac crest bone marrow. The results showed improved motor and sensory function in the group treated with conduct filled with stem cells [317]. A case report by Grimoldi et al. has shown a good functional outcome following the use of nerve conduit filled with skin-derived stem cells in a case of median and ulnar nerve defect [318].

Even though the results in these clinical trials are promising, there is still a long way to go towards the use of stem cells on a large scale, with the need to establish clear protocols in terms of the fabrication and delivery techniques to ensure patient safety and to achieve better functional outcomes [28].

Mesenchymal stem cell therapy has shown promise for treating peripheral nerve injuries, due to their ability to differentiate into neural cells and modulate the injury microenvironment. However, their clinical application remains limited by significant drawbacks such as infusion-related toxicity, the risk of tumorigenicity, and the reduced efficacy with aging. As a result, attention has shifted to MSC-derived exosomes, which exert therapeutic effects primarily through paracrine signaling rather than cell engraftment. Exosomes have demonstrated potential in promoting peripheral nerve regeneration in clinical settings with a favorable safety profile, including low immunogenicity, minimal adverse effects, and ease of storage. Nonetheless, challenges remain regarding the standardization of their manufacturing to ensure product consistency [186].

Another application with translational potential in clinical peripheral nerve repair is the use of electrical stimulation. Initially studied in animal models, the efficacy of electrical stimulation in the process of nerve regeneration has recently been demonstrated in human subjects as well. In 2015, Wong et al. published a study on the effects of electrical stimulation on sensory recovery in patients with complete digital nerve transections. Following surgical nerve repair, fine wire electrodes were implanted subcutaneously in 36 patients with similar injuries. Postoperatively, patients were randomly assigned to two double-blinded groups. The first group received continuous electrical stimulation at 20 Hz for one hour, while the control group received sham stimulation. The outcomes of both groups were assessed at 5 and 6 months, with overall better sensory recovery observed in patients who received active electrical stimulation [319].

There is compelling evidence supporting an increased rate of nerve regeneration in severe cases of carpal tunnel syndrome following surgical decompression and electrical stimulation. A study conducted by Gordon et al. quantified this effect through the postoperative placement of electrodes along the median nerve, proximal to the incision site, followed by electrical stimulation at 20 Hz for one hour. Patients who received electrical stimulation exhibited significant axonal regeneration at 6–8 months post-surgery compared to those treated with surgery alone. Furthermore, sensory conduction and terminal motor latency improved earlier in the electrically stimulated group than in the control group [320].

Similarly, Zhang X. et al. demonstrated the benefits of intraoperative electrical stimulation in the surgical management of cubital tunnel syndrome. Following surgical decompression, alternating high- and low-frequency electrical stimulation was applied for 15 min to the area most severely affected by compression. Outcomes were compared with those of a control group consisting of patients with cubital tunnel syndrome who underwent surgery alone. At 1 and 6 months postoperatively, significant improvement in symptoms was observed, with superior outcomes in both sensory and motor function in the electrically stimulated group compared to controls [321].

Effective rehabilitation is crucial after nerve repair, grafting, or transfer to optimize recovery. Early intervention focuses on controlled movement to prevent adhesions, stiffness, and contractures. Sensory and motor re-education play a key role in restoring function by promoting neural plasticity and reorganization. Successful outcomes depend on the patient’s active engagement in rehabilitation [8,322].

## 7. Conclusions

Peripheral nerve injuries present a considerable therapeutic challenge, requiring a customized approach to maximizing functional recovery. Successful nerve repair depends on multiple factors, including the severity of the injury, the length of the nerve gap, the condition of the surrounding tissue, and the available surgical and technological resources. Given the complexity of nerve regeneration, selecting the most appropriate repair method is important for achieving the best possible outcomes. Autografts remain the gold standard for nerve reconstruction due to their superior biocompatibility and ability to support axonal regeneration. However, they are associated with donor site morbidity, limited availability, and potential mismatches in nerve diameter and structure. To address these limitations, significant advancements have been made in nerve conduits, allografts, and regenerative medicine. Engineered nerve conduits, including synthetic and biological scaffolds, provide an alternative to autografts by guiding axonal growth across nerve gaps while reducing the risks associated with donor nerve harvesting. Meanwhile, allografts offer a viable option for larger defects, especially with the advent of decellularization techniques that minimize immune rejection. Additionally, regenerative medicine, encompassing stem cell therapy, growth factors, bioengineered scaffolds, biomolecules, pharmacological agents, and gene-based therapies, holds significant potential for enhancing nerve regeneration through the modulation of cellular signaling pathways and the optimization of the molecular microenvironment essential for effective axonal repair. These innovations aim to overcome the limitations of traditional nerve repair techniques, offering more effective, less invasive, and widely accessible solutions for peripheral nerve injuries. As research continues to advance, the integration of regenerative strategies with surgical techniques is expected to significantly improve functional recovery and quality of life for patients with nerve damage.

## Figures and Tables

**Table 1:** Classification of peripheral nerve injuries [2,9,12].

Classification System	Type/Grade	Description	Pathophysiology	Prognosis
Seddon Classification	Neurapraxia	Temporary conduction block	Localized demyelination without axonal disruption.	Complete recovery expected within days to weeks.
	Axonotmesis	Lesion in continuity with, axonal injury with intact connective tissue	Axonal damage with preservation of endoneurium, perineurium, and epineurium. Wallerian degeneration occurs distal to the injury.	Recovery possible without surgical intervention; axonal regrowth occurs at approximately 1 mm per day.
	Neurotmesis	Complete nerve severance	Total disruption of the nerve fiber, including axon and connective tissue.	If the nerve is completely transected, no recovery is possible without surgical repair.
Sunderland Classification	Grade I	Equivalent to neurapraxia	Conduction block without structural damage.	Full recovery expected within weeks.
	Grade II	Axonal disruption with intact endoneurium	Axonal damage with intact endoneurium; Wallerian degeneration occurs distal to the lesion.	Recovery occurs as axons regenerate along intact endoneurial sheaths.
	Grade III	Disruption of axon and endoneurium	Damage to axon and endoneurium, with perineurium intact; intrafascicular fibrosis may develop.	Partial recovery; misdirection of axons possible.
	Grade IV	Disruption of axon, endoneurium, perineurium	Preservation of only the epineurium; significant scarring impedes regeneration.	Recovery unlikely without surgical intervention.
	Grade V	Complete transection	Total severance of the nerve trunk, including all connective tissue structures.	Surgical intervention is essential; prognosis depends on timely and appropriate repair.
	Grade VI	Mixed injury (Mackinnon & Dellon)	Combination of injury severities within the same nerve.	Recovery varies; tailored surgical approaches may be necessary.

**Table 2:** Nerve conduits—properties and clinical applications [31,61,106,107,108,109,110,111,112,113].

Nerve Conduit Type	Biomaterial	Key Properties	Clinical Applications	Advantages	Disadvantages
Collagen Nerve Conduits	Type I collagen (from bovine or porcine sources)	Biodegradable and biocompatible Permeable Moderate mechanical strength	Repair of sensory and mixed nerves Nerve gaps up to 3 cm Commonly used in digital nerve injuries in the hand	Supports cell adhesion and migration Minimal immune response FDA-approved products available	Risk of disease transmission (animal-derived) Limited use in large nerve gaps
Polyglycolic Acid (PGA) Conduits	Synthetic polymer (example- Neurotube^®^)	Biodegradable High permeability Good flexibility	Repair of peripheral nerve gaps up to 3 cm Suitable for small-caliber nerves	Well-studied material Predictable degradation rate FDA-approved	Rapid degradation may lead to loss of support Degradation products can cause inflammation
Polycaprolactone (PCL) Conduits	Synthetic polymer	Biodegradable with slow degradation rate Good mechanical strength Low swelling	Repair of longer nerve gaps (up to 3 cm) Potential use in larger nerves due to mechanical strength	Long-term support for axonal growth Customizable properties through copolymerization	Slow degradation may impede tissue remodelling Limited clinical data compared to other materials
Chitosan Nerve Conduits	Natural polysaccharide derived from chitin	Biocompatible and biodegradable Antimicrobial properties Permeable	Experimental use in peripheral nerve injuries Potential for both sensory and motor nerve repair	Promotes nerve regeneration Low immunogenicity	Not widely available clinically Variability in material properties
Silicone Nerve Conduits	Non-biodegradable synthetic polymer	Biostable and non-degradable Flexible Impermeable (unless fenestrated)	Used in situations where long-term guidance is needed Historically used as a conduit before biodegradable options were available	Reusable and can be removed after regeneration Good mechanical protection	Requires second surgery for removal Risk of compression neuropathy due to non-degradability
Polyurethane Nerve Conduits	Synthetic polymer	Biodegradable Elastic and flexible Tunable permeability	Repair of small to medium nerve gaps Potential use in both peripheral and central nervous system injuries (experimental)	Mechanical properties similar to native nerve tissue Supports cell infiltration	Degradation products may cause inflammation Limited clinical use
Gelatin-Based Conduits	Natural protein derived from collagen	Biodegradable Good biocompatibility Permeable	Experimental applications in peripheral nerve repair Short nerve gaps	Supports cell adhesion and proliferation Easily modified with growth factors	Mechanical weakness Rapid degradation may not provide sufficient support
Hybrid Conduits (e.g., PCL/Collagen blends)	Combination of synthetic and natural materials	Tailored degradation rates Improved mechanical strength Enhanced biocompatibility	Repair of complex nerve injuries Potential for longer nerve gaps due to improved properties	Combines advantages of both materials Customizable to specific injury requirements	Complexity in manufacturing Regulatory approval challenges

Nerve conduits can be classified based on material composition in autograft-based conduits and synthetic nerve conduits.

**Table 3:** Key pathways involved in peripheral nerve regeneration [132].

Pathway	Effect
PI3K/Akt	Promotes cell survival, axonal elongation, and remyelination.
MAPK/ERK	Regulates neuronal differentiation and Schwann cell proliferation.
JNK/c-Jun	Controls neuronal plasticity and inflammatory responses.
RhoA/ROCK	Inhibits axonal outgrowth; its suppression enhances nerve regeneration.
JAK/STAT	Facilitates Schwann cell differentiation and myelin formation.
Nrg1-ErbB	Facilitates Schwann cell differentiation and myelin formation.

**Table 4:** Comparative analysis of techniques used for bridging a gap in peripheral nerves.

Nerve Repair Technique	Advantages	Disadvantages
Autogenous Nerve Grafting	- Biocompatible, no risk of immune rejection; - - Effective for bridging nerve gaps.	- Can cause sensory deficits at the donor site; - Risk of scarring and neuroma formation; - Requires an additional surgical incision; - Limited availability of donor nerves; - Less effective than tension-free primary nerve repair.
Nerve Transfers	- Avoids complications associated with nerve graft harvesting; - Closer proximity to target motor endplates enables faster reinnervation.	- Potential loss of function at the donor nerve site; - Donor muscle is no longer available for future muscle transfer.
End-to-Side Coaptation	- Useful for cases where only minimal sensory recovery is required.	- Does not support motor recovery unless donor axons are injured.
Nerve Allografts	- Readily available with an unlimited supply; - Can bridge large nerve gaps; - Eliminates donor site morbidity.	- Requires immunosuppression, which carries potential risks and side effects.
Nerve Conduits	- Easily accessible and eliminates donor site complications; - Facilitates nerve gap bridging; - Provides a protective barrier against scar tissue infiltration; - May promote local accumulation of neurotrophic factors.	- Outcomes are inconsistent; - Lacks essential components like laminin scaffolds and Schwann cells; - Only suitable for repairing short nerve gaps.

**Table 5:** Clinical indications and description of the main commercially available nerve conduits.

Product Name	Manufacturer	Type	Clinical Indications	Description
Avance^®^ Nerve Graft	Axogen^®^	Processed allograft	Used for bridging peripheral nerve defects.	A three-dimensional scaffold designed to bridge nerve gaps, composed of a decellularized and cleansed extracellular matrix that supports remodeling and nerve regeneration. It is offered in various lengths and diameters to accommodate different gap sizes and anatomical requirements [299].
Axoguard^®^ Nerve Connector	Axogen^®^	Porcine ECM conduit	Coaptation of severed peripheral nerves short gaps.	A porcine submucosa extracellular matrix proposed for the approximation and tensionless repair of severed peripheral nerves with less than a 5 mm gap, allowing for natural healing where cells incorporate into the ECM to form tissue similar to the nerve’s epineural connective tissue [300].
Axoguard ^®^ Nerve Protector	Axogen^®^	Porcine ECM wrap	Protect injured peripheral nerves without gaps, preventing scar tissue formation.	A porcine submucosa ECM surgical implant proposed for the separation and protection of injured nerves without gaps, preventing soft tissue adherence while allowing cell incorporation to remodel a separating tissue layer. It is suitable for nerves up to 40 mm [301].
NeuraGen^®^ Nerve Guide	Integra LifeSciences	Collagen conduit	Suitable for bridging small peripheral nerve gaps.	A resorbable, semi-permeable bovine, type I collagen-based tubular implant for the repair of peripheral nerve defects (of 3 cm or less), maintaining an interface between the nerve and surrounding tissue and serving as a conduit for axonal growth [302].
NeuraGen^®^ 3D Nerve Guide Matrix	Integra LifeSciences	Collagen conduit filled with collagen-glycosaminoglycan (C6S) matrix	Optimize environment for mid-gap nerve regeneration.	3D micro-architecture designed to mimic the natural structure of peripheral nerve tissue. The outer collagen conduit acts as a semi-permeable membrane, allowing the diffusion of small nutrient molecules into the guide while retaining larger molecules, such as nerve growth factors, within the conduit, promoting organized axonal regeneration. Addresses the nerve gaps of a maximum length of 3 cm, with diameters ranging from 1.5 mm to 7 mm [303].
NeuraWrap^®^ Nerve Protector	Integra LifeSciences	Collagen wrap	Encase the injured nerves, protecting against external compression and minimizing neuroma risk.	A resorbable bovine, type I collagen implant serving as an interface between the nerve and surrounding tissue through its porous outer layer and a semi-permeable internal membrane that allows for diffusion of small molecules while retaining nerve growth factors. Can wrap nerves from 3 mm to 10 mm in diameter with a maximum length of 4 cm [304].
Neuroflex^®^ Connector	Stryker	Collagen conduit	Severed nerves across joints, treatment of symptomatic or painful neuromas.	A resorbable type I collagen-based tubular matrix with corrugated side walls, it is highly flexible and kink-resistant—bends up to 60° without occlusion, maintains conduit shape during joint motion, and reduces risk of compression or collapse in dynamic areas. It is available in various diameters with a length of 2.5 cm [305].
NeuroMatrix ^®^ Connector	Stryker	Collagen conduit	Severed nerve injuries in straight anatomical path.	A resorbable, type I collagen-based conduit designed to provide structural support and tensionless repair across straight gaps. The only available length is 2.5 cm [306].
NeuroMend^®^ Collagen Wrap	Stryker	Collagen wrap	Protect crushed or compressed nerves from mechanical irritation.	A resorbable, semi-permeable collagen wrap that provides a protective sheath around injured peripheral nerves, helping to reduce suturing and supporting regeneration. It is suitable for nerves ranging from 2.0 mm to 12.0 mm in diameter, being available in 2 lengths (2.5 cm and 5.0 cm) [307].
Neurolac^®^ and Neurolac^®^ Thin wall(TW)	Polyganics	Synthetic conduit made from poly(DL-lactide-ε-caprolactone) (PLCL)	Short nerve gaps repair, prevent neuroma formation/	These bioabsorbable conduits are designed for the reconstruction of peripheral nerve gaps up to 20 mm, commonly in the hand and wrist. The TW version has a thinner wall structure to improve flexibility and allow easier handling or needle penetration when needed [308].

## Data Availability

No new data were created.
